# MULocDeep: A deep-learning framework for protein subcellular and suborganellar localization prediction with residue-level interpretation

**DOI:** 10.1016/j.csbj.2021.08.027

**Published:** 2021-08-18

**Authors:** Yuexu Jiang, Duolin Wang, Yifu Yao, Holger Eubel, Patrick Künzler, Ian Max Møller, Dong Xu

**Affiliations:** a Department of Electrical Engineering and Computer Science, Bond Life Sciences Center, Columbia, MO, USA; b Institute of Plant Genetics, Leibniz University Hannover, Hannover, Germany; c Department of Molecular Biology and Genetics, Aarhus University, Forsøgsvej 1, DK-4200 Slagelse, Denmark

**Keywords:** Protein localization, Mechanism study, Deep learning, Experimental benchmark datasets, Web server

## Abstract

Prediction of protein localization plays an important role in understanding protein function and mechanisms. In this paper, we propose a general deep learning-based localization prediction framework, MULocDeep, which can predict multiple localizations of a protein at both subcellular and suborganellar levels. We collected a dataset with 44 suborganellar localization annotations in 10 major subcellular compartments—the most comprehensive suborganelle localization dataset to date. We also experimentally generated an independent dataset of mitochondrial proteins in *Arabidopsis thaliana* cell cultures, *Solanum tuberosum* tubers, and *Vicia faba* roots and made this dataset publicly available. Evaluations using the above datasets show that overall, MULocDeep outperforms other major methods at both subcellular and suborganellar levels. Furthermore, MULocDeep assesses each amino acid’s contribution to localization, which provides insights into the mechanism of protein sorting and localization motifs. A web server can be accessed at http://mu-loc.org.

## Graphical abstract

**Figure:**
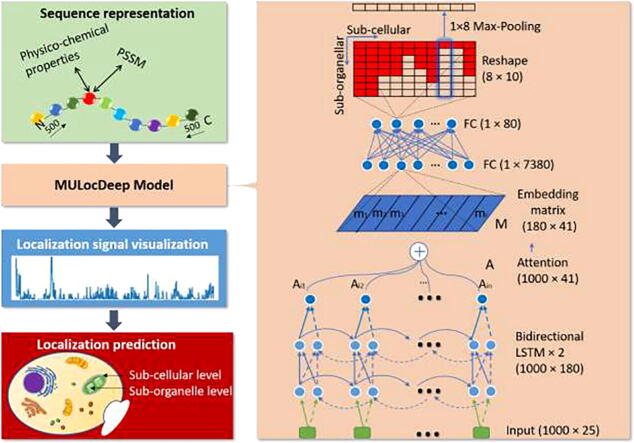

## 1 Introduction

In eukaryotic cells, proteins perform diverse functions governed by the compartments or organelles in which those proteins are located. The aberrant localization of proteins is often associated with diseases, such as Alzheimer’s disease, metabolic disorders, and cancers [1], [2]. The mechanism of protein localization is complex, and it is often controlled by many factors, including signal peptides, protein trafficking, protein–protein interaction, folding, and alternative splicing [2], [3]. Among them, protein localization guided by targeting peptides is the most common mechanism [4], which includes pre-sequences and internal signals [5], [6]. Pre-sequences are found at the N- or C-terminus of a protein sequence with an enrichment of charged or hydrophobic amino acids, while internal signals are in the middle of sequences. The process by which the precursor proteins are directed to the target organelle is only partially understood [5] and the number of experimentally identified targeting peptides (especially internal signals) is not much. According to the annotation in UniProt (release 2020_05), out of the reviewed 20,394 human proteins, 7348 have localization annotation with experimental verification, while only 3608 (17.7%) proteins have known targeting peptides. Furthermore, very limited sub-organellar compartment localization data are available. According to our recent research, out of the 16,213 human proteins in the 10 organelles on which we focused, 5882 have experimentally verified organellar localization annotation, while only 3518 have experimentally verified sub-organellar localization annotation. Targeting peptide and sub-organelle data for non-human species are much scarcer. Despite the development of technologies such as mass spectrometry and fluorescence tagging, experimental identification of protein subcellular/sub-organelle localizations is still time-consuming and labor-intensive [7], [8], [9]. Thus, computational methods can play an important role in this area.

Computational protein localization prediction is mainly through machine learning approaches to extract features from training samples. Before deep learning, these features are predefined and will not change during the training process. For example, WoLF PSORT [10] converts a protein’s amino acid sequence into features like sorting signals, amino acid composition, and motifs for training a k-nearest neighbor (KNN) classifier. TPpred3 [11] detects the targeting signal in the N-terminal region of a protein based on a support vector machine (SVM) classifier. Predotar [12] applies a neural network to identify proteins targeting the endoplasmic reticulum (ER), mitochondria, and plastids in plants by N-terminal targeting signals. TargetP [13], [14] also uses neural networks to discriminate proteins destined for mitochondrion, chloroplast, and the secretory pathway based on the N-terminal sequence information. Some methods also take advantage of homology information and Gene Ontology (GO) annotations [15] if available. Representative methods include LocTree3 [16], SherLoc2 [17], MultiLoc2 [18] , and YLoc [19]. These methods have better performance when reliable annotations of homologous proteins are available. More recently, deep learning methods have been explored in protein localization prediction. For example, DeepLoc [20] uses a convolutional neural network (CNN) and long short-term memory (LSTM) to give a prediction of 10 subcellular protein localizations. Deep neural networks were used in our previous MU-LOC method [21], in which features including amino acid frequency, sequence profile, and gene co-expression were used to predict whether or not a plant protein is mitochondrial. The latest version of TargetP (v2.0) [22] applies bidirectional LSTM to predict thylakoid transit peptides.

Although several methods have achieved good prediction results on specific protein localization cases, these methods still face limitations and many unsolved problems. Most of the methods focus on the prediction of protein localization at the subcellular level. Although there are some predictors for specific suborganelle localizations [22], [23], [24], [25], a systematic suborganelle localization prediction tool at the whole-cell scale is still missing. Furthermore, protein localization is a multi-label problem, i.e., one protein may be found in several different compartments in a cell. Some efforts have been made in multi-label prediction [24], [26], but current deep learning-based methods simplify the protein localization prediction as a one-label classification problem in which each protein can only be predicted at a unique compartment—which is not the case for 15–20% of all proteins (Fig. S1). In addition, there is room to increase the deep learning model’s interpretability for characterizing localization signals. For example, both TargetP 2.0 [22] and DeepLoc [20] attempted to identify strong contributing sequence factors to localization using the attention mechanism [27], [28]. However, TargetP 2.0 considers only the first 200 amino acids near the N-terminus of a protein but cannot detect localization signals in other parts of a protein. DeepLoc addresses this problem by taking as many as 500 amino acids from each terminus of a protein, but the interpretation resolution for the contribution to localization is not as high as TargetP 2.0 because of the usage of CNN layers.

In this paper, we propose a multi-label protein localization framework named MULocDeep that covers 10 main subcellular localizations and 44 suborganellar localizations. A matrix layer is designed to capture the intrinsic hierarchical relationships between organelles and their subcompartments, enabling our method to make predictions at both levels simultaneously. Similar to the TargetP 2.0 model, the MULocDeep framework uses the Long Short Term Memory (LSTM) [29] and multi-head self-attention [27], to extract biological features that contribute to localizations at the single amino acid resolution. Some of these features match the current knowledge of protein sorting signals, while there are novel discoveries that could provide some new insights. This paper also includes an experimental study, in which the mitochondrial proteomes of three species, Arabidopsis cell cultures, potato tubers, and bean roots, were extracted and identified (*Mito3* dataset). We also systematically collected a dataset from the UniProt database [30], containing proteins of eukaryotic species in 44 suborganellar compartments in 10 subcellular localizations with experimental evidence (*UniLoc* dataset). Evaluations using the above datasets show that overall, MULocDeep outperforms other major methods at both subcellular and suborganellar levels. The datasets themselves can be used as benchmarks for methods developed by others. The datasets, the source code, and the web server of our study are all publicly available.

## 2 Material and methods

### 2.1 Datasets

#### 2.1.1 The three-species mitochondrial proteome dataset (Mito3).

This dataset contains the mitochondrial proteome extracted from three plant species (*Arabidopsis thaliana, Solanum tubers, and Vicia faba*). The identification of these proteins is part of our results and is introduced in Section 3.1. Here we present its statistics and the processing of it as an evaluation dataset. A total of 8002 proteins from these three species were identified (2818 from *Vicia faba*, 2470 from *Solanum tubers*, and 2414 from *Arabidopsis thaliana*). After assigning these proteins to their Arabidopsis orthologues by pairwise BLAST searches of the underlying sequence datasets, 4778 unique mitochondrial proteins were identified. To use this dataset for localization classifier evaluation at the subcellular level (predicting if a protein is mitochondrial or not), we balanced it by collecting 8002 plant proteins that were labelled as non-mitochondrial in the UniProt database (release 2020_04). The proteins in this dataset were not used in training the model. To perform a more rigorous test, we applied blastp [31] to align the *Mito3* dataset against the *UniLoc-train-40nr* dataset. If a hit has an alignment coverage higher than 80% of the shorter sequence and a 40% sequence identity or 10^-5^ E-value, it will be removed. The remaining sequences in *Mito3* were kept and formed *Mito3-40nr* (1929 positive samples and 1450 negative samples).

#### 2.1.2 Uniprot dataset (UniLoc)

The protein sequence and localization annotations were downloaded from the UniProt database release 2020_04 [30] with the following constraints: 1. The existence code must be protein or transcript level. 2. Proteins must be complete and fragment proteins were removed. 3. Proteins that are encoded in mitochondrion, chloroplast, and plastid were removed. 4. Proteins that do not start with Methionine or have a sequence length of fewer than 40 amino acids were removed. One protein can have more than one localization annotation, and we chose suborganelle localizations with more than 50 proteins and ignored others in this study. Finally, 44 suborganelle localizations under 10 subcellular localizations remained. This is the most comprehensive multi-label dataset for protein localization annotations down to the suborganelle level to date. For convenience, we annotate the subcellular level as lv1, and the suborganellar level as lv2. From the data we collected (*UniLoc* dataset), we picked the protein samples that only have subcellular localization annotations, with (lv1_exp) or without (lv1_noexp) experimental evidence code (ECO:0000269). The number of samples are 11,435 and 38,443, respectively. Then, from the *UniLoc* dataset, we picked the protein samples that have suborganellar localization annotations, with (lv2_exp, 11,204 samples) or without ECO:0000269 (lv2_noexp, 32,303 samples). The main reason why we included protein samples without ECO:0000269 is that some of the suborganellar classes have so few samples when only including proteins with ECO:0000269. For each of the 10 classes in lv1_exp, 15% of the protein samples were used as testing (choosing protein samples created after 2018 in UniProt first; if less than 15% of the corresponding class, then selecting randomly). A similar way was used to select the testing sample for each of the 44 suborganellar classes from lv2_exp. The testing samples at both levels construct the final testing dataset (*UniLoc-test,* 4532 samples), and in this way, we make sure that the localization annotations of testing samples are experimentally verified and the testing samples cover every class. Specific samples were further selected from *UniLoc-test* in certain cases. For example, *UniLoc-sub* is composed of proteins in *UniLoc-test* that have suborganellar localization annotations, particularly, submitochondrial proteins (176 samples), subGolgi proteins (46 samples) and subchloroplast proteins (82 samples). *UniLoc-multi* (687 samples) is composed of proteins in *UniLoc-test* that have at least two different subcellular localization annotations. *UniLoc-single* (3847 samples) is composed of proteins in *UniLoc-test* that only have one subcellular localization annotation.

The remaining data samples (experimentally verified or not) were combined as the training dataset (*UniLoc-train, 88,853*). Using the testing dataset, a non-redundant training dataset (*UniLoc_train_40nr*) was created by removing redundant proteins in the training dataset using blastp. Specifically, we align each of the proteins (query) in the *UniLoc_train* against *UniLoc_test*. If a query protein finds a hit with sequence identity higher than 40% or E-value 10–5, and the alignment covers more than 80% of the shorter sequence of the query and its hit, the query will be removed. The resulting *UniLoc-train-40nr* has 33,100 proteins. The same *UniLoc-test* dataset was used to evaluate the model performance after removing the redundancy effect. We did not use a sequence identity threshold lower than 40%, since it would lose too many samples and leave not enough samples for training in many classes. The statistics of samples in all the classes in *UniLoc* dataset are shown in Table S1.

#### 2.1.3 External datasets

We used the datasets provided by other methods to train and test a variant model for a rigorous evaluation of methodology. Two datasets (*SM424-18 and SubMitoPred*) are from the DeepMito method which focuses on the prediction of sub-mitochondrial protein localization. One dataset (*DeepLoc dataset*) is from the DeepLoc method which predicts 10 main localizations at the subcellular level. The *SM424-18* dataset was derived from the UniProt database (release 2018_02). They filtered the proteins by selecting all non-fragment protein sequences with evidence at the protein level for experimentally determined subcellular localization in one of the four sub-mitochondrial compartments: outer membrane, intermembrane space, inner membrane and matrix. They further reduced the redundancy using the CD-HIT program so that 424 mitochondrial proteins remained sharing at most 40% sequence identity. The *SubMitoPred* dataset was derived from the UniProt database release 2014_10. The protein selection criteria were: full-length proteins greater than 50 residues, single experimental sub-mitochondrial localization, and internal redundancy reduced at 40% sequence identity using CD-HIT. The dataset comprises 570 mitochondrial proteins distributed in the same four compartments as in the SM424-18 dataset. Both datasets were split into folds, 10 for the SM424-18 dataset and 5 for the SubMitoPred dataset. The category details can be found in Table S2. The *DeepLoc dataset* was extracted from the UniProt database, release 2016_04. It contains proteins from experimentally annotated 10 main compartments in eukaryotic cells. A total of 13,858 proteins were obtained and the training and testing samples are marked. The number of proteins in each compartment of the DeepLoc dataset is shown in Table S3.

### 2.2 MULocDeep framework and the workflow

**Fig. 1:**
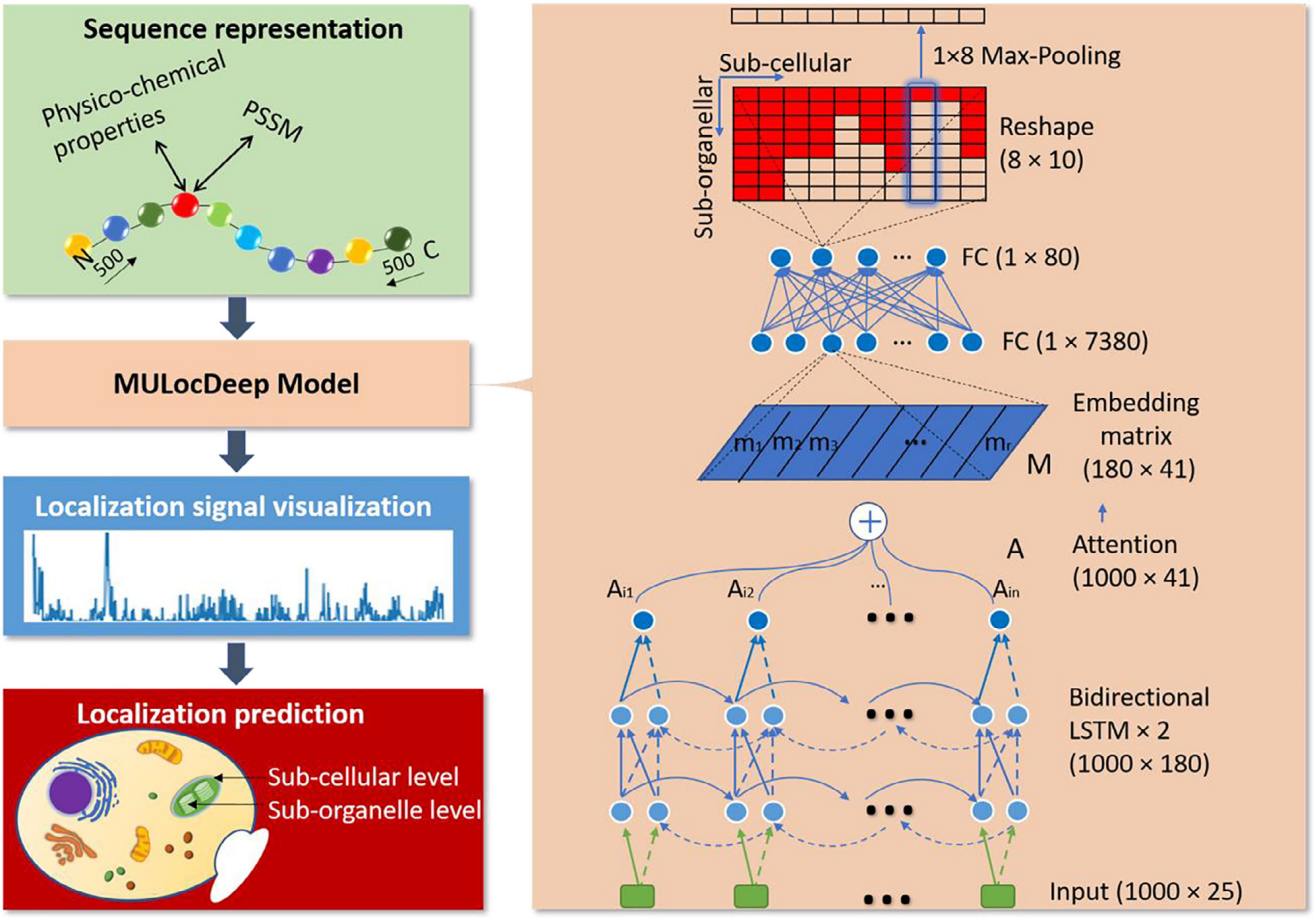
MULocDeep workflow and neural network architecture. The workflow is composed of four steps: (1) Protein sequence representation, (2) the MuLocDeep model training, (3) localization signal visualization, and finally (4) localization prediction. The details of the neural network architecture are displayed in the right panel.

The workflow of our framework is presented in Fig. 1. Protein sequences with known localization information were collected, processed, and fed into our deep learning model for training. During the training process, the output of the “attention” layer was extracted separately for sorting signal interpretation and visualization. Finally, the trained model was used to predict localization for new proteins. A description of the MULocDeep model is shown in the right panel in Fig. 1. The input layer was composed of encoded protein sequences with a fixed length of 1000 amino acids. Each amino acid was encoded as a 25-dimension vector (see the Methods section for encoding details). The input layer was followed by two layers of bidirectional LSTM (29), which ensured that every amino acid received a signal from both sides. Two such layers were stacked to give the model the ability to fit complex high-order functions. The sequence length remained unchanged after the bidirectional LSTMs, while only the encoding dimension was changed to 180. Then a multi-head self-attention layer [27] was applied (“A” in Fig. 1). The embedding matrix (“M” in Fig. 1) was derived as the weighted sum by multiplying the attention layer with the output from the bidirectional LSTM. The attention itself was also an output to assess the contribution of each amino acid to localization. The embedding matrix was flattened into a 7380 (180X41) long vector, and then fully connected with an 80-dimensional dense layer, which was further reshaped into an 8-by-10 matrix. Each column of the matrix represents a major subcellular localization (10 organelles) and each element under the column represents a suborganelle category. In our UniLoc dataset, some organelles contained up to eight suborganellar localizations. For other organelles that had fewer suborganelle localizations, the empty slots in the matrix were padded with zeros. When processing a new sample, the predicted value in the matrix was used for the suborganelle prediction. Then a 1 × 8 max-pooling layer was applied to the matrix so that the highest predicted value of a suborganelle localization was used as the prediction score of the corresponding organelle localization. Only the suborganellar prediction scores were compared with the thresholds, i.e., a suborganelle with a prediction score above the threshold would trigger the prediction of corresponding organelle localization (see the Methods section for threshold determination details). In this way, we could perform multi-label predictions at both subcellular and suborganelle levels and keep the results consistent. This matrix design also enables MULocDeep to utilize protein samples that only have subcellular localization annotations. The MULocDeep model was trained using the *UniLoc-train* dataset, which was divided into 8 folds. We performed cross-validation by using 7 folds as training and 1 fold as evaluation each time. Table S4 shows the cross-validation results at the suborganelle level with different prediction thresholds. When training the MULocDeep model, we tried different strategies to tune the hyperparameters and tested their impact on the performance. The details of the training process and hyperparameter configuration are described in the Methods below.

### 2.3 Protein sequence representation

An encoded amino acid contains two parts. The first 5 dimensions come from the first five eigenvectors of a comprehensive list of 237 physical–chemical properties for each amino acid [32]. As we did in the domain boundary prediction study [33], these 5-number descriptors can represent each amino acid for computational efficiency while maintaining almost all the information. The last 20 dimensions come from the position-specific scoring matrix (PSSM) profile of a protein. A protein’s PSSM profile is usually generated through a multiple sequence alignment against a large database. Some methods try to accelerate the process by searching a relatively small database first, and if no hit is found then use a large database instead [20], [34]. We further shortened this process by two steps, first by scanning the Swissprot [35] using PSI-blast [36]. The Swissprot database is a much smaller database than UniProt, yet most of the proteins that we studied find hits. If no hits were retrieved, in the next step, the BLOSUM62 encoding [37] was applied directly, which did not take any search time. In this way, we saved much computational time without a significant performance decrease (see Results). Since the length of proteins varies, we fixed the protein-encoding length at 1000 AA. If a protein exceeded this length, the first 500 amino acids from N-terminus and the last 500 amino acids from C-terminus were preserved and combined. If a protein had a shorter sequence, we padded it to 1000 AA at the end and masked the padding part for the following calculation.

### 2.4 Parameter tuning and neural network training

The hyperparameters in the models are determined through a Bayesian optimization process. These hyperparameters include the hidden dimensions, the number of heads in attention, regularizers, dropout rates, *etc*. The MULocDeep model is an ensemble of eight “sub-models” derived from an 8-fold cross validation. Each of these eight sub-models was optimized individually and the hyperparameters of the sub-model that achieved the highest accuracy were set as the final optimum. Table S5 lists the hyperparameter optimization results for all eight sub-models in the cross validation. The hyperparameter configuration in sub-model 1 was selected as the final optimum. Therefore, in the MULocDeep model, all the eight sub-models used the same final optimized hyperparameters. We also tested the performance of an ensemble of sub-models where each sub-model 1) using its own optimized hyperparameters, 2) using the same, but not the final optimized, hyperparameters (select two from seven non-final optima), or 3) using the same random hyperparameters. The results are shown in Table S6. It turns out that the difference in performance among various ensemble models was insignificant, except for a notably poor performance by using the randomly selected hyperparameters. We also conducted an experiment to test the performance of individual models using the final optimum hyperparameters. The results are listed in Table S7. Comparing the results in Table S6, the ensemble models generally have a better performance than individual models.

The *UniLoc-train* dataset was divided into 8 folds. Eight models were trained where each of them used 7 folds as training and 1 fold was left for evaluation. The final MULocDeep model is the ensemble of eight models, and the prediction of a protein is the average of predictions from these eight models. All these models used the same final optimized hyperparameters. To train each of these models, the lv1_train and lv2_train in the *UniLoc-train* dataset were utilized iteratively. Specifically, we trained them using the samples with only subcellular localization labels (lv1_train) for 1 epoch, and then trained another epoch using the samples with suborganelle localization labels (lv2_train). This process was repeated 80 times for each of the eight models. When a training sample had both suborganelle and subcellular (inferred from suborganelle) annotation, each element in the matrix (Fig. 1) yielded a loss using a binary cross-entropy loss function after a sigmoid activation function (Lost 1). The maximum prediction score under each organelle (each column in the matrix in Fig. 1) was extracted and went through another binary cross-entropy (Lost 2). If a training sample only had the subcellular localization information, the Lost 1 was not used, only the Lost 2 was calculated after the Max-Pooling operation. The pseudo code for the training process is provided in Supplementary Note 1.

The training process was written using the Keras package (version 2.3.0) and run using an NVIDIA GeForce RTX 2080 Ti GPU. The training time for the MULocDeep model was roughly 2 min for one epoch.

The thresholds for predicting suborganelle localizations were determined from cross-validation results by *UniLoc-train* at the suborganelle level with different prediction thresholds (if the prediction output is above the threshold, a positive label is predicted), which is shown in Table S4. The default threshold is 0.5, and we tuned it in a way that favors positive predictions (high recall) based on the results in Table S4. In particular, we only tuned the thresholds in the range below 0.5 to achieve the highest MCC. The same threshold determination process of MULocDeep was also applied to the training of the non-redundant model.

### 2.5 Bayesian optimization

We formulate the accuracy “ACC” as the objective function and it is a function of all the hyperparameters. A Gaussian process was used as the surrogate model to approximate the objective function. We used the expected improvement (EI) as the acquisition function, which directs sampling to areas where an improvement over the current best observation is likely. The acquisition jitter, which trades off exploitation (high objective) and exploration (high uncertainty) was set as 0.05.

Since the optimization process would take a long time, we used training samples provided by the DeepLoc method instead of our own *UniLoc-train* dataset. Since DeepLoc also focuses on the same 10 subcellular localizations of eukaryotic proteins, we assume the distribution of data should be similar between the two datasets. We divided these samples into 8 folds using CD-hit [36] and the sequence identity between proteins in different folds was below 40%. Then, an 8-fold cross validation was performed. Each hyperparameter has a searching space (shown in Table S5). During the cross validation, 7 folds were used for training a sub-model under one specific hyperparameter configuration for 40 epochs. The remaining fold evaluated the accuracy in each epoch. The highest accuracy on the validation fold during the 40 epochs was recorded as the accuracy for this hyperparameter configuration. In total, 150 hyperparameter combinations were tested. Fig. S2 shows the accuracy of the testing process. The hyperparameter configuration which achieved the highest accuracy among the 150 combinations was used as the optimized configuration for this sub-model (shown in Table S5). Thus, the optimization process would run 40 (test one specific configuration) * 150 (150 configurations to test in total) * 8 (8-fold cross-validation), which is 48,000 epochs in total. Finally, each of the eight sub-models had its own optimized hyperparameter configuration and the corresponding accuracy was achieved. The configuration with the highest accuracy was selected as the final optimized configuration that was used for the MULocDeep model and its variant models.

### 2.6 Multi-head self-attention

The multi-head self-attention [27] uses the overall semantics of the whole sentence formed by multiple components in a sentence. So, multiple hops of attention are needed to focus on different parts of the sentence. Our method borrows this idea and sets the number of heads equals 41 (derived from the hyperparameter tuning). The final weight of each amino acid is the average of the 41 weights. Then we could analyse if any “important parts” of a protein sequence are responsible for the protein localization. The attention matrix A is calculated as Eq. (1)

(1) $$A=softmax(W_{s2}tanh\left(W_{s1}H^{T}\right))$$

where *H* is the 1000-by-180 embedding sequence output from bidirectional LSTM. $W_{s1}$ is a weight matrix with a shape of 369-by-180. $W_{s2}$ is a matrix of parameters with a shape 41-by-369. The attention matrix *A* is returned separately for interpretation. The sequence embedding *M*, calculated as the weighted sum by multiplying *A* and *H* (Eq. 2), is also returned for further prediction.

(2) M=AH

When training the models, we applied the penalization term *P* below [27]

(3) $$P={\left\|(AA^{T}-I)\right\|}_{F}^{2}$$

where *A* is the attention matrix, *I* is an identity matrix, ${\left\|∙\right\|}_{F}$ stands for the Frobenius norm of a matrix. We multiple the penalization term with an attention regularizer and added the product to the model’s loss. The loss reaches the maximum if two attention vectors are identical, and it is the minimum when the attention vectors are orthogonal to each other. Thus, by using this penalization term, we encourage the attention vectors to concentrate on different parts of a protein sequence.

### 2.7 Attention visualization

To visualize the attention for one protein, the average of the protein’s attention matrix was calculated along the dimension of “heads”, which is 41 in our model. A 1000-long vector is left and ready for visualization.

To visualize the attention of a group of proteins that belong to the same category, different methods are applied based on the following two scenarios:

(1) For analyzing attention at termini, we simply align the first 50 or the last 50 amino acids one by one from left to right or right to left depending on if it is N-terminus or C-terminus. For each position, the frequency of amino acids and the weight of attention (average along with the number of “heads” and proteins) are obtained. Then we used the R package “ggseqlogo” [38] to visualize the attention.
(2) For analyzing attention in the middle of proteins, we kept the entire protein sequence and ranked the amino acid in it based on their attention weights. We selected the top 5 amino acids, each of them combining the surrounding 20 amino acids (10 window size at each side) to form a segment. A final segment was obtained by concatenating all segments by a string of “X” with the same length of window size. All segments belonging to the same class were analysed by the GLAM2 [39], a tool in the MEME Suite 5.1.0 that can discover variable-length, gapped motifs.

### 2.8 Evaluation criteria

We used accuracy (ACC), Matthew’s correlation coefficient (MCC) [40], recall, precision, area under the receiver operating characteristic curve (ROC_auc), and area under precision & recall curve (P&R_auc) to evaluate our method and compare it with others. For unbalanced datasets, measurements such as ACC, recall and precision would introduce bias and overestimate a method’s performance. MCC considers true and false positives and negatives, and is generally regarded as a balanced measure even if the classes are of very different sizes [41]. The definitions of ACC, MCC, recall and precision are listed in Eqs. 4–7:

(4) $$\mathrm{ACC}=\frac{\mathrm{TP}+TN}{\mathrm{TP}+TN+FP+FN}$$

(5) $$\mathrm{MCC}=\frac{\mathrm{TP}*TN-FP*FN}{\sqrt{\left(TP+FP)(TP+FN)(TN+FP)(TN+FN\right)}}$$

(6) $$\mathrm{recall}=\frac{\mathrm{TP}}{\mathrm{TP}+FN}$$

(7) $$\mathrm{precision}=\frac{\mathrm{TP}}{\mathrm{TP}+FP}$$

where *TP, FP, TN, FN* are true positive, false positive, true negative and false negative predictions, respectively. Measurements such as MCC, recall, precision and ACC can be used in binary prediction cases.

When training a MULocDeep model, the loss function used for both suborganellar and subcellar levels is the binary cross-entropy as defied in Eq. (8):

(8) $$L=-\frac{1}{C}\sum_{i=1}^{C}{[y}_{i}∙\log\left(p\left(y_{i}\right)\right)+\left(1-y_{i}\right)∙log\left(1-p\left(y_{i}\right)\right)]$$

where *C* is the number of localization categories and $y_{i}$ is the label of category *i*. This loss function enables MULocDeep to predict multi-label localizations. When training a MULocDeep variant model to compare with single-label prediction methods, we used the categorical cross-entropy as the loss function as defined in Eq. (9):

(9) $$L=-\sum_{i}^{C}t_{i}\log\left(Softmax\left(P\right)\right)$$

where *C* is the number of localization categories and *P* is the prediction vector for all the categories.

## 3 Results

In this part, we first present the mitochondrial proteome we experimentally extracted from three species. Then we evaluate the MULocDeep model by comparing it with other methods. We demonstrate the effectiveness of MULocDeep in interpreting the contribution of each amino acid to localization prediction. Some of these important amino acids can match well-known protein sorting peptides or signals. Finally, we briefly introduce the key features of the MULocDeep web server.

### 3.1 Mitochondrial proteome from three species

We experimentally extracted the mitochondrial proteome from *Arabidoposis thaliana*, *Solanum tubers*, and *Vicia faba*. The whole process can be roughly divided into three steps. First is the mitochondria isolation from each species. Then, the mitochondrial proteins of the three species were identified and quantified using shotgun mass spectrometry according to Thal et al. [42]. Finally, the generated raw files were analysed with the Proteome Discoverer software (Thermo Fisher Scientific, Dreieich, Germany) using the Mascot (Matrix Science, London, UK) search engine against in-house protein sequence databases. The technical details of the process are introduced in Supplementary Note 3. The Vicia proteins (identified as Medicago proteins) and Solanum proteins were assigned to their Arabidopsis orthologues through pairwise BLAST searches of the underlying proteome datasets. The e-value and word size for a protein assignment were set to 10^-172^ and 3 for Solanum tuberosum, and 10^-178^ and 3 for *Medicago truncatula* proteins, respectively. The Arabidopsis hit with the highest score was the chosen orthologue. From this, a non-redundant list containing the original Arabidopsis hits as well as potato and Vicia orthologues was produced, whose entries represent candidates for mitochondria located proteins. The identified proteins from the three species with the respective Arabidopsis orthologues are shown in Supplementary Data 1–3. We are aware of the existence of co-purifying proteins from other organelles within each of the three species, which were not removed. However, removing these ‘contaminants’ is just as wrong as including them. As a hypothetical case, if a dually localized protein (e.g., plastids and mitochondria) was first reported to be present in the plastid, then the database registers it as belonging there, although it belongs just as much in the mitochondria. It would be wrong to remove it from the mitochondrial proteome. Another example is the groups of proteins (e.g., cytoplasmic ribosomes, cytoskeleton components, ER proteins, glycolytic proteins) that interact with proteins in the outer mitochondrial membrane. These proteins will therefore co-purify with mitochondria at least under some conditions. However, it is questionable to treat them as contaminants.

### 3.2 Comparison of performance in localization prediction

Firstly, we compared the protein localization prediction of MULocDeep with other available methods on four benchmark datasets provided by us, the *Mito3*, the *UniLoc-sub, the UniLoc-single* and the *UniLoc-multi* datasets. We summarized a list of localization classifiers regarding their scope (target localizations), availabilities (web server or local tool) and the performance on these datasets. We have investigated many other methods, but they were excluded from the comparison because they were either unavailable, not working properly at the time of test, or only accept a single sequence for web submission. The *Mito3* dataset was used to evaluate the performance of different classifiers for mitochondrial proteins (Table 1, upper part). The *UniLoc-sub* dataset was used to test the suborganellar localization prediction for Golgi, mitochondrion, and chloroplast (Table 1, middle part). The *UniLoc-single* dataset was used to compare with DeepLoc for the 10 main subcellular localization predictions (Table 1 lower part). Since the *UniLoc-test* dataset mostly consisted of proteins created after 2018 in the UniProt database, we assume that for most of the comparing methods, the same protein sequence did not appear in their training sets. Some new methods, e.g., DeepMito, may not guarantee that, but this would be to the advantage of these methods instead of ours since no sequence in the *UniLoc-test* dataset was used in training our model. Hence, the comparison in Table 1 can be regarded as an evaluation from the tool perspective, which mimics the actual usage that cares about the prediction performance instead of the homology issue. To evaluate the generalization of our method, the performance of MULocDeep model trained using the non-redundant training set (*UniLoc-train-40nr*) is also shown in Table 1 separated by a slash.

**Table 1:** Evaluation and Comparison of Protein Localization Prediction Methods.

**Mitochondrion localization prediction using the *Mito3* dataset:**										
Method	Scope	AVAIL	Subcellular	Suborganellar	Assessments					
					ROC_auc	P&R_auc	MCC	Recall	Prec	Acc
MULocDeep	1–10	W&L	Mitochondrion	/	0.74/0.69	0.79/0.77	0.39/0.31	0.30/0.29	0.94/0.90	0.64/0.57
MULoc(21)	4	W&L		/	**0.78**	**0.82**	**0.42**	**0.52**	0.85	0.67
DeepLoc [20]	1–10	W&L		/	0.70	0.63	0.39	0.35	0.76	**0.75**
TargetP v5 [22]	3,4,7	W&L		/	0.72	0.79	0.23	0.13	**0.97**	0.50
MitoFates [44]	4	W&L		/	0.63	0.71	0.22	0.15	0.90	0.50
SherLoc2 [17]	1–11	W		/	0.68	0.73	0.22	0.18	0.85	0.51
MultiLoc2 [18]	1–11	W&L		/	0.68	0.73	0.22	0.15	0.90	0.50
Predotar [12]	4,6,7	W		/	0.60	0.68	0.24	0.21	0.87	0.53
**Suborganellar localization prediction using *UniLoc-sub* dataset:**										
MULocDeep	1–10	W&L	Mitochondrion	Inner membrane	**0.83**/0.75	**0.74/0.70**	0.41/0.37	**0.92**/0.78	0.55/0.58	0.65/0.67
				Outer membrane	0.71/0.49	**0.67**/0.27	**0.67**/0.18	0.59/0.13	**0.86**/0.50	**0.91**/0.82
				Matrix	**0.88**/0.76	**0.81**/0.53	**0.71**/0.42	**0.86/0.64**	0.72/0.51	**0.89**/0.77
				Intermem. space	0.83/0.58	**0.56**/0.16	0.38/0.15	0.31/0.50	0.56/0.16	0.91/0.71
			Golgi apparatus	Trans-Golgi	0.72/0.59	**0.92**/0.85	0.38/0.26	0.63/0.23	**0.92/1.00**	0.67/0.41
				Cis-Golgi	**0.81**/0.49	**0.58**/0.26	**0.48**/0.06	**0.64**/0.09	**0.58**/0.33	**0.80**/0.74
			Plastid (chloroplast)	Membrane	**0.54**/0.36	**0.51**/0.25	**0.45/0.12**	**0.38**/0.21	**0.82**/0.21	**0.79/0.54**
				Stroma	**0.80/0.60**	**0.76/0.43**	**0.63/0.13**	**0.67/0.52**	**0.82/0.40**	**0.84**/0.59
				Thylakoid lumen	**0.86/0.82**	**0.49/0.29**	**0.52/0.30**	**0.50/0.67**	**0.60/0.22**	**0.94**/0.80
				Thylakoid mem.	**0.87/0.72**	**0.84/0.68**	**0.38/0.30**	**0.86**/0.59	**0.50/0.53**	**0.65/0.67**
DeepMito [23]	4	W&L	Mitochondrion	Inner membrane	0.78	0.66	**0.48**	0.82	**0.63**	**0.73**
				Outer membrane	**0.80**	0.57	0.62	**0.65**	0.72	0.89
				Matrix	0.78	0.59	0.60	0.61	**0.76**	0.86
				Intermem. space	**0.85**	0.53	**0.65**	**0.75**	**0.63**	**0.93**
SubGolgi v2 [43]	8	W	Golgi apparatus	Trans-Golgi	**0.77**	**0.92**	**0.47**	**0.85**	0.88	**0.80**
				Cis-Golgi	0.77	0.45	0.47	0.63	**0.58**	**0.80**
TetraMito [45]	4	W	Mitochondrion	Inner membrane	0.61	0.55	0.17	0.52	0.51	0.59
				Outer membrane	0.71	0.59	0.45	0.46	0.62	0.85
				Matrix	0.62	0.29	0.06	0.50	0.26	0.55
Schloro [46]	7	W	Plastid (chloroplast)	Membrane	0.43	0.26	−0.09	0.29	0.23	0.51
				Stroma	0.50	0.32	0.00	0.00	0.00	0.67
				Thylakoid lumen	0.50	0.07	0.00	0.00	0.00	0.92
				Thylakoid mem.	0.53	0.37	0.03	0.65	0.36	0.47
**Subcellular localization prediction using *UniLoc-single* dataset:**										
MULocDeep	1–10	W&L	Nucleus	/	**0.97**/0.94	**0.93**/0.85	**0.81**/0.68	**0.91/0.87**	0.82/0.69	**0.93**/0.86
			Cytoplasm	/	**0.94**/0.86	**0.83**/0.66	**0.71**/0.53	**0.83**/0.74	**0.74**/0.59	**0.89**/0.81
			Extracellular	/	**0.99**/0.98	**0.92**/0.83	**0.87/0.82**	**0.92/0.89**	**0.86**/0.80	**0.97/0.96**
			Mitochondrion	/	**0.98**/0.94	**0.92**/0.82	**0.87**/0.74	0.84/0.70	**0.92/0.82**	**0.98**/0.96
			Cell membrane	/	**0.96**/0.91	**0.89/0.76**	**0.77**/0.64	**0.86/0.76**	**0.76**/0.65	**0.94**/0.90
			ER	/	**0.96**/0.87	**0.82**/0.50	**0.80**/0.48	**0.75**/0.41	**0.88**/0.62	**0.98**/0.95
			Plastid	/	**0.99**/0.94	**0.86**/0.68	**0.83**/0.71	**0.82**/0.64	**0.85**/**0.79**	**0.99/0.99**
			Golgi apparatus	/	**0.95**/0.89	**0.71**/0.49	**0.70**/0.44	**0.67**/0.57	**0.75**/0.37	**0.98**/0.96
			Lysosome	/	**0.97**/0.80	**0.63**/0.10	**0.66**/0.14	**0.50**/0.10	**0.87**/0.20	**0.99/0.99**
			Peroxisome	/	**0.98/0.94**	**0.75**/0.53	**0.75/0.54**	**0.57**/0.38	**1.00/0.78**	**0.99/0.99**
DeepLoc	1–10	W&L	Nucleus	/	0.96	0.91	0.78	0.85	**0.83**	0.91
			Cytoplasm	/	0.90	0.78	0.64	0.75	0.72	0.86
			Extracellular	/	0.97	0.85	0.81	0.84	0.83	0.95
			Mitochondrion	/	0.97	0.89	0.82	**0.86**	0.82	0.97
			Cell membrane	/	0.92	0.80	0.72	0.67	0.87	0.93
			ER	/	0.93	0.75	0.70	**0.75**	0.69	0.96
			Plastid	/	0.96	0.76	0.74	0.81	0.68	0.98
			Golgi apparatus	/	0.93	0.66	0.66	0.62	0.72	**0.98**
			Lysosome	/	0.88	0.32	0.35	0.47	0.28	0.98
			Peroxisome	/	0.93	0.55	0.52	0.54	0.52	**0.99**

The upper part of the table uses our *Mito3* dataset to evaluate the performance of the mitochondrial protein prediction; the middle part uses the *UniLoc-sub* dataset to evaluate the performance at the suborganelle level prediction; and the lower part uses the *UniLoc-single* dataset to evaluate the performance at the subcellular level. Availability (AVAIL) is either through a web server (W) or a local tool (L). The prediction scope includes compartments in: 1. nucleus; 2. cytoplasm; 3. extracellular; 4. mitochondrion; 5. cell membrane; 6. endoplasmic reticulum; 7. plastid/chloroplast; 8. Golgi apparatus; 9. lysosome/vacuole; 10. peroxisome; 11. plasma membrane. Criteria of assessment are ROC_auc (area under the receiver operating characteristic curve), P&R_auc (area under precision & recall curve), MCC (Matthew’s correlation coefficient), recall, precision, and accuracy. Localization categories with less than 6 samples were removed. The performance after the slash is from the MULocDeep method trained using non-redundant dataset (*UniLoc-train-40nr*). It was evaluated using the same test datasets, except the upper part of the table, where non-redundant MULocDeep model was evaluated using *Mito3-40nr*.

Among the six measurements of the performance, the ROC_auc (area under the receiver operating characteristic curve) and P&R_auc (area under precision & recall curve) are the most important criteria from the method perspective as they reflect accuracies in a continuous range of thresholds for a binary prediction while the other measurements are affected by the chosen thresholds. In the upper part of Table 1, MULoc, a mitochondrion-specific method developed by our lab has the highest score in most of the measurements. Except for this, MULocDeep has more than half of the measurements better than any other method in a pair-wise comparison. Especially, the ROC_auc and P&R_auc are consistently higher in MULocDeep than others. At the suborganellar level prediction (Table 1 middle), MULocDeep is consistently better in predicting subchloroplastic proteins. It is also generally better in predicting *cis*-Golgi network proteins, mitochondrion matrix and outer membrane proteins. DeepMito [23] is better for the prediction of the mitochondrion intermembrane space proteins and SubGolgi v2 [43] is better in *trans*-Golgi network protein prediction. It is noted that all the better methods than MULocDeep are organelle-specific, which suggests tuning models for a single organelle may have some advantages. In the lower part of Table 1, MULocDeep achieved a higher score in most of the measurements than DeepLoc. It is worth mentioning in Table 1 that MULocDeep’s performance using the non-redundant training set did not drop significantly compared to the model using the redundant training set in terms of ROC_auc and P&R_auc which indicates a good generalization of MULocDeep.

The *UniLoc-multi* dataset was used to compare with pLoc-mEuk [47], which is a subcellular localization prediction method for multi-label eukaryotic proteins (i.e., predicting multiple labels simultaneously). All the proteins in the *UniLoc-multi* dataset have more than one localization annotation. The comparison result for the multi-label localization prediction is shown in Table 2. We used the exact match as the criterion to evaluate the overall performance of each method, and MCC, recall, precision, and accuracy to evaluate individual classes. According to Table 2, MULocDeep, either the redundant or non-redundant model, has significantly better individual scores than pLoc-mEuk in almost every case.

**Table 2:** Comparison of Multi-label Protein Localization Prediction Performance.

*UniLoc-multi* dataset:						
Method	Localization	MCC	Recall	Prec	ACC	Exact match
MULocDeep	Nucleus	**0.59/0.53**	**0.66/0.62**	**0.99/0.97**	**0.76/0.72**	**243**/144(**35%/**21%)
	Cytoplasm	**0.36**/0.21	**0.68/0.59**	**0.95**/0.92	**0.70/0.61**	
	Extracellular	**0.60/0.29**	**0.60/0.40**	**0.60/0.22**	**0.99/0.99**	
	Mitochondrion	**0.47**/0.23	**0.40**/0.20	**0.60**/0.33	**0.96/0.95**	
	Cell membrane	**0.74/0.51**	**0.73/0.54**	**0.85/0.64**	**0.93/0.86**	
	ER	**0.70**/0.41	**0.53**/0.22	**1.00/0.87**	**0.94/0.90**	
	Golgi apparatus	**0.73/0.33**	**0.58/0.32**	**0.98**/0.47	**0.96/0.90**	
	Lysosome	**0.64/0.23**	**0.47/0.12**	**0.89/0.50**	**0.99/0.98**	
pLoc-mEuk	Nucleus	0.38	0.57	0.89	0.65	161(23%)
	Cytoplasm	0.28	0.52	**0.95**	0.57	
	Extracellular	0.09	0.20	0.05	0.97	
	Mitochondrion	0.28	0.26	0.36	0.94	
	Cell membrane	0.36	0.34	0.58	0.83	
	ER	0.43	0.32	0.70	0.89	
	Golgi apparatus	0.33	0.26	0.54	0.90	
	Lysosome	0.11	0.05	0.25	0.97	

Criteria are Matthew’s correlation coefficient (MCC), recall, precision (Prec), accuracy (ACC), and exact match. The exact match means that there is no difference in all the organelle labels between the predicted ones and the experimental ones. The best score in each sub-category is shown in bold. Localization categories with less than 6 samples were removed. The performance after the slash is from the MULocDeep method trained using non-redundant dataset (*UniLoc-train-40nr*).

To evaluate different approaches from the method perspective under a rigorous condition, we created a variant of the MULocDeep model. We used the variant model to compare with different methods individually at both subcellular and suborganellar levels. The details of the variant model are provided in Supplementary Note 2. At the suborganellar level, only a few methods have provided clearly separated datasets for training and testing, which makes it difficult to make a fair comparison. Here we compared with DeepMito [23], a recently published deep learning method for sub-mitochondrial protein localization prediction. A variant model was trained using the same *DeepMito* datasets provided by the DeepMito paper. The output layer was a 4-dimensional vector representing four target compartments (outer membrane, inner membrane, intermembrane space and matrix) in mitochondria as in DeepMito. Processing the data as in the DeepMito method, the *SM424-18* dataset and the *SubMitoPred* dataset were split into 10 and 5 folds, respectively. The comparison was based on the MCC of different compartments from the cross validation as reported in the DeepMito paper [23]. MULocDeep performed better than DeepMito for every mitochondrial compartment in both datasets (Table 3).

**Table 3:** Comparison of Method Effectiveness between MULocDeep and DeepMito.

Dataset	Method	Feature/CV method	MCC(O)	MCC(I)	MCC(T)	MCC(M)
SM424-18	DeepMito	SEQ	0.17	0.15	0.13	0.07
		PROP	0.17	0.07	0.22	0.13
		PSSM	0.51	0.47	0.42	0.57
		SEQ + PROP	0.16	0.07	0.55	0.09
		PSSM + PROP	0.46	0.47	0.53	0.65
	MULocDeep	PSSM + PROP	**0.53**	**0.59**	**0.59**	**0.67**
SubMitoPred	SubMitoPred	RS	0.42	0.34	0.19	0.51
	DeepMito	RS	0.45	0.68	0.54	0.79
	DeepMito	CL	0.42	0.60	0.46	0.76
	MULocDeep	RS	**0.67**	**0.76**	**0.67**	0.79

The comparison is at the sub-organellar level. Four target compartments are “O”: Outer membrane, “I”: Inner membrane, “T”: Intermembrane space, and “M”: Matrix. Features used include one-hot encoding residue (SEQ), physico-chemical properties (PROP), and position-specific scoring matrix (PSSM). Cross-validation (CV) methods include randomly splitting the dataset (RS) and confining local similarity into the same cross-validation set (CL). The assessment is based on Mathew’s correlation coefficient (MCC).

For protein localization prediction at the subcellular level, the DeepLoc dataset was originally used to compare between DeepLoc and eight other methods [20]. The results show that the DeepLoc method outperformed other methods in terms of both accuracy and Gorodkin value [48]. To demonstrate the effectiveness of our method in predicting protein localization at the subcellular level, like what we did for the sub-mitochondrial prediction, the variant model with ten target localizations (nucleus, cytoplasm, extracellular, mitochondrion, cell membrane, endoplasmic reticulum, plastid, Golgi apparatus, lysosome, and peroxisome) was trained using the same training dataset and tested on the same testing dataset that was used for DeepLoc. To further reduce the bias resulting from inappropriate implementation or parameter configuration, we directly quote the performance reported in the DeepLoc paper [20] and show the prediction results in Table 4.

**Table 4:** Comparison of Method Effectiveness between MULocDeep and DeepLoc.

Localization	MCC from MULocDeep	MCC from DeepLoc
Nucleus	0.725	**0.784**
Cytoplasm	0.549	**0.608**
Extracellular	0.896	**0.907**
Mitochondrion	**0.823**	0.812
Cell membrane	0.696	**0.732**
Endoplasmic reticulum	0.602	**0.654**
Plastid	**0.901**	0.883
Golgi apparatus	**0.464**	0.414
Lysosome	**0.208**	0.194
Peroxisome	**0.412**	0.321

The comparison is at the subcellular level. Both methods were trained using the training samples in the *DeepLoc* dataset and tested using the testing samples in the *DeepLoc* dataset.

As shown in Table 4, MULocDeep has comparable performance in terms of prediction accuracy (each method has 5 out of 10 classes better than the other method). One reason is that our method used a much smaller database for PSSM profile generation (see Methods) than DeepLoc. A benefit of that is that MULocDeep is much faster than DeepLoc. It takes approximately 4.2 s to run MULocDeep per protein, which is a prediction speed more suitable for online usage than DeepLoc, which takes approximately 30 s per protein.

### 3.3 Attention weight interpretation and visualization

MULocDeep can not only make accurate localization predictions but also indicate the contribution of each amino acid in localization and suggest localization motifs. This is achieved by attentive embedding through assigning higher weights to specific parts of a protein sequence. We assume that the regions with higher attention weights are more likely to contribute to the localization. When using a high resolution of attention, it is possible to predict sites and motifs relevant to protein localization. For example, the peptide cleavage site could be predicted directly from an amino acid level attention [22]. Our method provides interpretable results for all the 44 types of suborganelle localizations as far as 500 amino acids from each terminus.

**Fig. 2:**
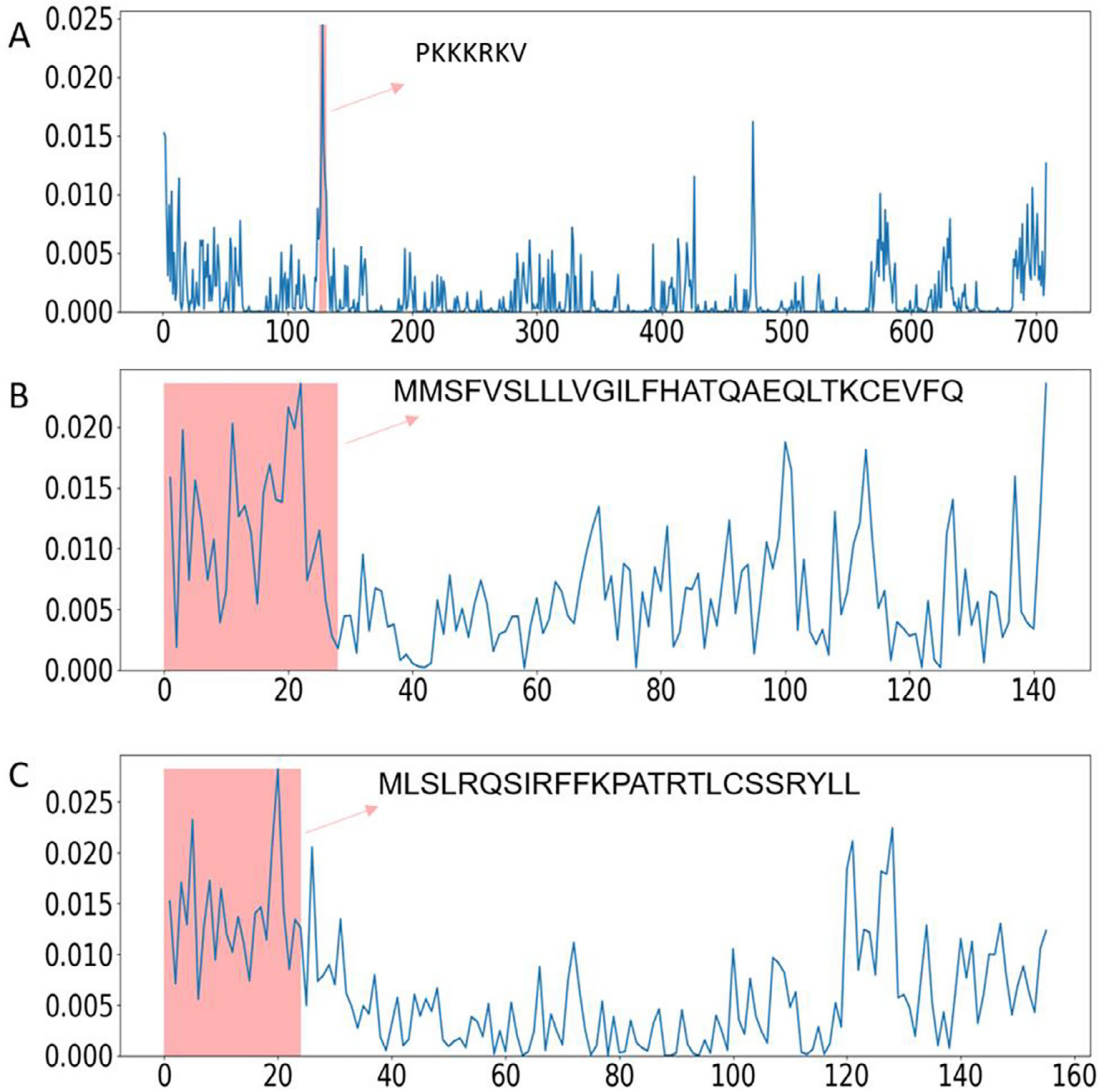
Visualization of attention weights for (A) SV40 large T antigen (P03070), (B) lactalbumin (P09462), and (C) cytochrome oxidase subunit 4 (P04037). The x-axis presents the sequence position from the N-terminus to the C-terminus, and the y-axis presents the value of attention weights. The region of the known sorting motif is highlighted in peach and labelled with the sequence of known localization signals.

Firstly, we used several proteins with known localization signals as cases to demonstrate the ability of attention weights for indicating the contribution of each amino acid in localization. The proteins are: SV40 large T antigen (P03070) located at the nucleus, with the known signal motif “PKKKRKV” in the middle of the protein sequence; lactalbumin (P09462) located at the secreted pathway, with a known signal peptide “MMSFVSLLLVGILFWATEAEQLTKCEVFQ” at the N-terminus; and COX4 (P04037) located in the mitochondrial inner membrane, with the known transit peptide “MLSLRQSIRFFKPATRTLCSSRYLL” at the N-terminus [49], [50], [51]. When using these proteins as input, MULocDeep predicted the localization correctly for all three proteins. We also obtained the attention weights for each protein along the sequence, as shown in Fig. 2, indicating that the high attention regions match the corresponding known signal motifs of the proteins.

**Fig. 3:**
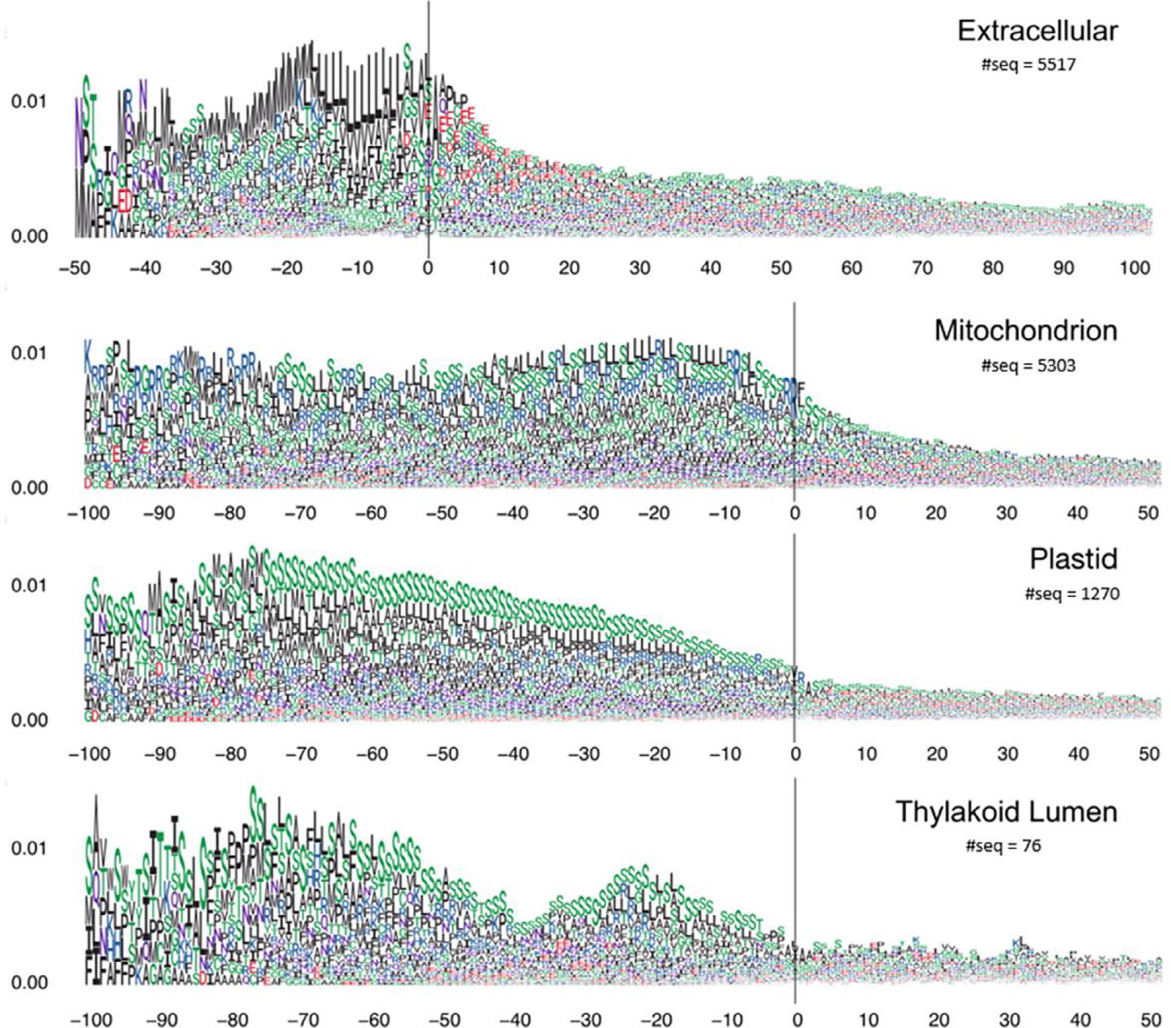
The attention weight vs. sequence position by aligned N-terminal sequences at the cleavage site for proteins localized at extracellular, mitochondrion, plastid and thylakoid lumen. The vertical lines indicate the cleavage sites. For extracellular proteins, the range covers 50 AAs before the cleavage cite and 100 AAs after the cleavage cite. For the other three classes of proteins, the range covers 100 AAs before the cleavage cite and 50 AAs after the cleavage cite. The number of protein sequences in each class is shown.

Next, we investigated the attention weights in terms of groups of proteins from the same subcellular compartments and the same suborganelle compartments. Firstly, we visualized the attention weights of proteins from ten subcellular compartments (Figs. S4 to S6). Among them, the localization of proteins in extracellular, mitochondrial, plastid, and thylakoid lumen (Fig. S4) are believed to be controlled by signal peptides near the protein N-terminus [22]. Comparing to other localizations (Figs. S5 and S6), the signals near the N-termini of proteins in Fig. S4 have higher attention weights, more over-represented amino acid patterns, and maintain at high levels for longer sequence segments. Our result is consistent with TargetP in detecting the N-terminal sorting signals using attention weights [13], [22]. These N-terminal sorting signals are often proteolytically removed at the cleavage cites after the protein reaches the final destination. We then aligned the weighted sequences of these four types of proteins at the cleavage site. The cleavage site annotation was obtained from the UniProt database. The attention visualization result is shown in Fig. 3. An immediate decrease in the attention weight is observed after the cleavage site for the proteins from all four subcellular localizations. This indicates that the high attention weights near the N terminus are mostly contributed by signal peptides and transit peptides.

Looking at the attention weights at the termini of proteins in all ten subcellular localizations (Figs. S4-S6), it is apparent that the attention weight increases towards the termini in all cases although more so at the C-terminus. We, therefore, wondered if there is a terminus attention bias introduced by the MULocDeep method. We did a control experiment to test for such a terminus bias on the proteins. For each localization, we randomly shuffled the order of amino acids for each protein sequence. Then we plotted the attention weights aligned at the termini for these four localizations and indeed found some terminal bias (Fig. S7). We can use this to distinguish true and false-positive signals, which all give high attention weights near the termini. As shown in Fig. S7, false-positive signals are characterized by a gradual decrease from the terminus and the absence of dominant amino acids at each residue position. In contrast, the features of true signals are: amino acids in each position are more conserved, the high attention signal lasts relatively longer and sometimes the highest attention weight appears as a ‘bump’ away from the termini (in all four localizations shown in Fig. S4). Hence, although the terminus attention bias exists, the attention weights in MULocDeep may add values to illustrate biologically significant signals. We then divided the attention weights from the real protein sequences of the four protein classes in Fig. S7 by the attention weights from the randomly shuffled protein sequences. The result is shown in Fig. S8. For protein classes that do not have an N-terminal signal (nucleus, cytoplasm), their attention weights are similar to the random one and the attention ratio is near 1. While for the protein classes that are known to have an N-terminal signal peptide, e.g., extracellular and mitochondrion, there is a region that the ratio is much higher than 1.

**Fig. 4:**
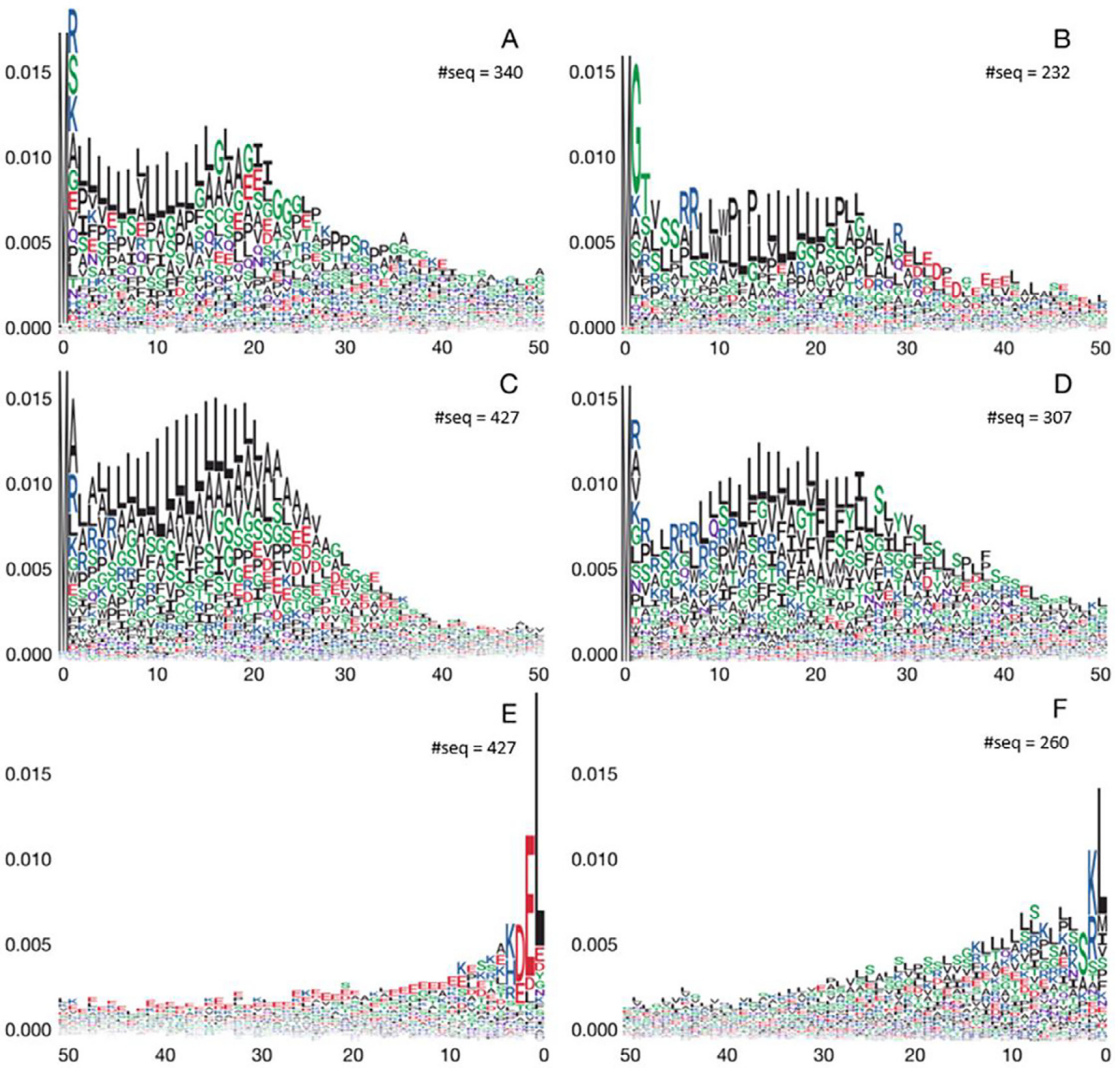
Attention weights of 50 amino acids vs. sequence position near the termini at the sub-organellar level, which suggests potential localization signals. (A) N-terminus of cytoplasmic granule. (B) N-terminus of cell surface. (C) N-terminus of endoplasmic reticulum lumen. (D) N-terminus of Golgi apparatus and Golgi stack membrane. (E) C-terminus of endoplasmic reticulum lumen. (F) C-terminus of peroxisome proteins.

Since the attention weights of a subcellular localization reflect the average of attention weights of its suborganellar localizations, there still could be suborganellar localizations that have strong N-terminal or C-terminal sorting signals even though the subcellular sorting signal is not obvious. We, therefore, show the attention weights of proteins at the suborganellar localizations in nucleus, cytoplasm, cell membrane, endoplasmic reticulum, Golgi apparatus, lysosome, and peroxisome (Figs. S9-S15). We found several suborganelle localizations with strong signals near the termini. These signals are shown in Fig. 4, including N-terminal signal peptides in proteins from cytoplasmic granule (Fig. 4A), cell surface (Fig. 4B) and endoplasmic reticulum lumen (Fig. 4C), and perhaps also in Golgi apparatus membrane and Golgi stack membrane (Fig. 4D), which all resemble the endoplasmic reticulum signal peptide observed for extracellular proteins in Figs. S4 and S6. A C-terminal KDEL/HDEL signal in the endoplasmic reticulum lumen proteins (Fig. 4E), a C-terminal SRL/SKL/SRM signal for the peroxisome (Fig. 4F), and a less clear sequence for the peroxisome membrane (Fig. S15) [52], [53] are observed.

**Fig. 5:**
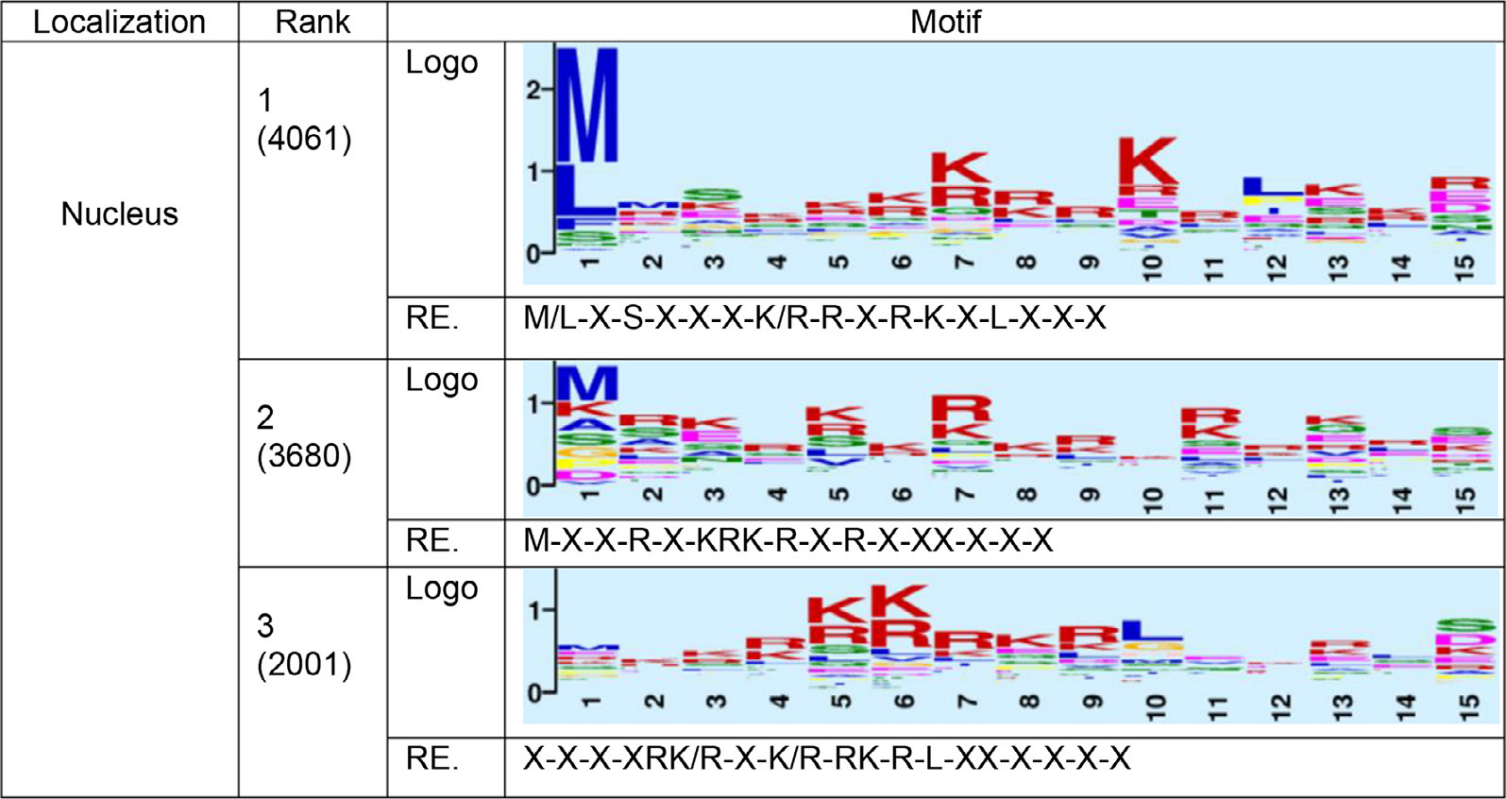
The top three GLAM2 results for segments from nucleus proteins. For each result, its rank, score, sequence logo and the regular expression (RE) of the motif are given.

Besides the signals near the termini, we also analyzed the attention weights in the middle. We aligned sequences in the same way as we did at the termini, but no signal was found at all. A likely reason is that the signals in the middle do not appear in the same position for different proteins. Thus, the sorting signal in the middle of protein sequences was analyzed and visualized with the help of the GLAM2 tool [39] in the MEME Suite, which can discover over-represented, position-independent motifs in protein sequences (see Methods for details). A well-known internal signal is the nuclear localization signal (NLS). The visualization in Fig. 5 using nuclear proteins was obtained by setting the “initial columns” (initial number of aligned columns in the motif) equal to 15, the “maximum columns” (upper bound on the number of aligned columns in the motif) equal to 30 while other parameters remain the default in the GLAM2 online tool. The classical NLS pattern includes stretches of 4–5 Lys or Arg (e.g. KKKK) [49], which was readily recognized by GLAM2 (Fig. 5).

We carried out similar GLAM2 analyses on proteins from six more subcellular localizations (Figs. S16-S21) and found a number of well-defined internal signals where the signal found in the cytoplasm deserves special mention (Fig. S16, motif rank 2) – A L/I/VxxxxxxL/V/I/F motif, which is known to be a nuclear export signal [54].

**Fig. 6:**
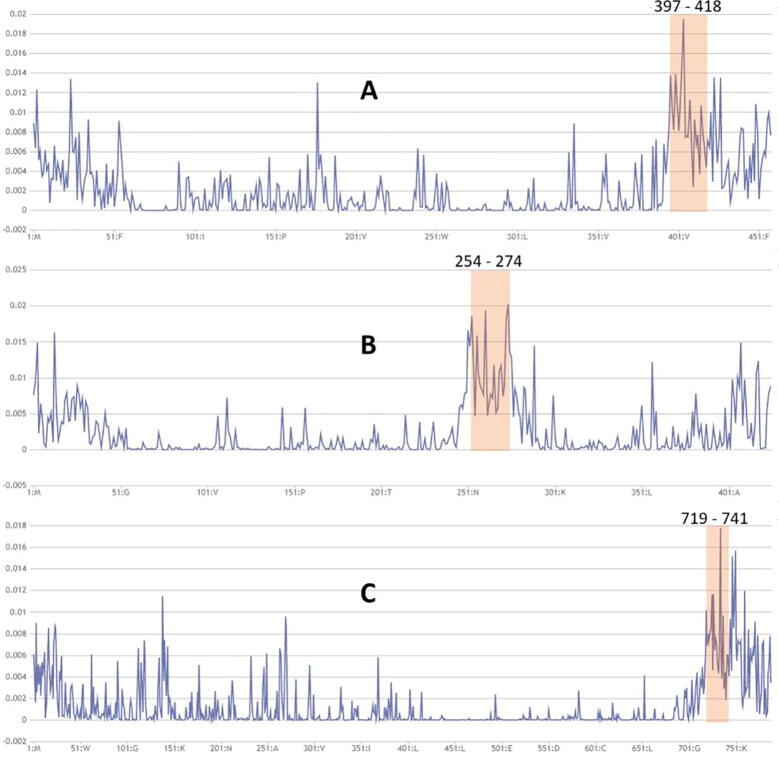
Visualization of attention weights for (A) CD4_HUMAN (P01730), (B) TNR16_RAT (P07174), and (C) ITB3_HUMAN (P05106). The x-axis presents the sequence position from the N-terminus to the C-terminus, and the y-axis presents the value of attention weight. The region of the known transmembrane region is highlighted in orange and labelled with the “start – end” positions. (For interpretation of the references to colour in this figure legend, the reader is referred to the web version of this article.)

The interpretation of attention weights does not have to be limited to target peptides. If a localization class is well predicted by our model, some features must have been learned, which are probably reflected by high attention weights. It is worth checking if those features have any biological meaning. We did such evaluations on membrane proteins, specifically, transmembrane proteins that span the membrane only once. The alignment results of the selected transmembrane protein attention weights and the known transmembrane position annotations are shown in Fig. 6. For all three cases, the peak of their attention weights matches the transmembrane regions annotated in the Uniprot databases. This suggests that the membrane interaction regions play important roles in protein localization, possibly through membrane protein-mediated transport [55]. Even though we did not use such information to train the MULocDeep model, it learns this feature automatically to help the localization prediction. This indicates a broad interpretation ability by MULocDeep.

### 3.4 Application of MULocDeep_tool to the human proteome

**Fig. 7:**
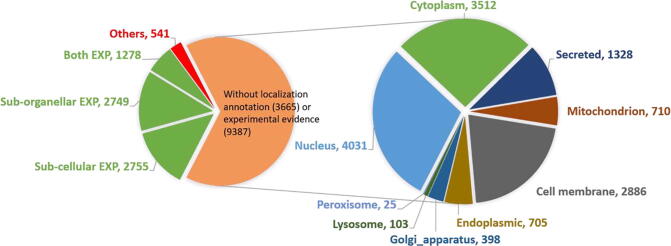
The human proteome localization pie chart. The left pie chart is based on the protein localization annotation, including with or without annotation, and with or without experimental evidence (ECO: 0000269). The green part in the left pie chart are proteins with experimentally verified localization annotations (ECO: 0000269). “Others” include those proteins contain annotations not in any of the 10 major sub-cellular or 44 sub-organellar localization annotations in MULocDeep, or have different localizations for different protein isoforms. The right pie chart is based on the distribution of prediction for the proteins in the orange part in the left pie chart. (For interpretation of the references to colour in this figure legend, the reader is referred to the web version of this article.)

We applied MULocDeep_tool in the human proteome and drew pie charts of statistics in Fig. 7. The human proteome data is collected from the UniProt database release 2020_04 [30]. Totally, 20,375 reviewed human proteins were collected. The green part in the left pie chart in Fig. 7 containing proteins with experimentally verified localization annotations (ECO: 0000269) is further divided based on if the experimental evidence is at the subcellular level, suborganellar level, or both. Since subcellular localization can be inferred from suborganellar annotation, one can consider all the proteins in the green part to have subcellular level experimental evidence. For the proteins without experimental evidence or without localization annotation at all (proteins in the orange part in the left pie chart), we combined them and applied MULocDeep_tool. The right pie chart is drawn based on the prediction results. More than half the proteins are predicted to be localized in the nucleus and cytoplasm. This result is consistent with the conclusion in Thul *et al* [7], which was obtained by mapping 12,003 human proteins at a single-cell level using immunofluorescence microscopy. Furthermore, according to Fig. 7, the number of human mitochondrial proteins is 1347 (combining the proteins with experimental evidence and the proteins predicted by MULocDeep_tool). This number matches the conclusion in Calvo *et al* [56], which estimated 1100–1400 distinct proteins in the human mitochondrial proteome. Thus, the prediction results in Fig. 7 reflect the true localization distribution in human and demonstrates the ability of MULocDeep_tool for proteome-wide annotation.

### 3.5 The MULocDeep web server

We developed a user-friendly website (http://www.mu-loc.org/) to make the application of the MULocDeep_tool more accessible. Every page comes with a “help” button, which explains how the specific page works. Every user has his or her personal workspace in our database in which they can manage their jobs conveniently. Many web servers offer protein localization prediction. Our website is unique in that this is the only web server that can predict all 44 suborganellar localizations within 10 main subcellular compartments. To the best of our knowledge, it is also the only web server that provides an amino acid level interpretation and visualization. Users can use this web server as a protein localization prediction tool or a hypothesis generator with regard to protein sorting signal motifs.

## 4 Discussion and conclusions

In this paper, we present “MULocDeep”, a deep learning method for protein localization prediction (shown in Fig. 1). The core of the model is the bidirectional LSTM to handle protein sequence information and the multi-head self-attention to assign weights to each amino acid of a sequence for interpretation. Some methods [20] added CNN layers before LSTM layers and reported high performance but at the cost of resolution for residue-level interpretation in contribution to protein localization. Here, we discard CNN to pursue a residue-level interpretation resolution for more biological insight.

To maximize prediction accuracy, we used a Bayesian optimization method to determine the hyperparameters. However, our experiments showed that the performance was insensitive to the selection of hyperparameters (Table S6). This indicates that our model is robust. The selected configuration of hyperparameters remained the same for a variant model and the MULocDeep_tool, instead of optimizing a new set of hyperparameters with the specific data, which could have yielded slightly better results.

Evaluations of different studies were conducted from both perspectives of method and tool. Representative methods at both subcellular and suborganellar levels were compared with a variant MULocDeep model, which was trained and tested with their own data. We also provide several benchmark datasets with the experimentally extracted mitochondrial proteome from three species (*Mito3* dataset) and a comprehensive dataset for 44 suborganelle protein localization from the UniProt database (*UniLoc* dataset). Note that even though the DeepLoc dataset was claimed to be split the training and testing using PSI-CD-HIT with 30% sequence identity, we found that 44% of testing samples can find a sequence in the training set that has a sequence identity above 30%. Many samples in testing have very high sequence identity with samples in training: 2.2% have sequence identity above 80%; 0.79% have sequence identity above 90%. This is, to some extent, caused by the heuristic nature of the algorithm of PSI-CD-HIT. However, this makes the DeepLoc dataset not a suitable dataset for method generalization evaluation. Our UniLoc dataset consists of UniLoc-train and UniLoc-test, as well as UniLoc-train-40nr where the sequences in UniLoc-train were removed if they have a sequence identity above 40% with a sequence in UniLoc-test using blastp (see Methods section). Thus, even though the identity threshold we used in blastp is higher (40% versus 30% in deeploc using PSI-CD-HIT), the actual resulting dataset is much more rigorous in terms of non-redundancy. The *Mito3-40nr* is the non-redundant version derived by removing proteins that have sequence identities above 40% against the *UniLoc-train-40nr* dataset. These benchmark datasets were used for a comprehensive evaluation of localization classifiers at both subcellular and suborganellar levels (Table 1). The results indicate a superior performance and a good generalization ability of MULocDeep.

The attention weight mechanism in the MULocDeep model can detect sorting signals at the protein termini and in the middle of protein sequences. The interpretation was validated by matching the known transit peptides for proteins located in the nucleus, extracellular, mitochondria, plastid, and thylakoid lumen (Fig. S4). Particularly, plastid and thylakoid lumen proteins have a high attention peak at position 2 from the N terminus, which is enriched in alanine. A region enriched in serine follows. The signal peptide in extracellular proteins apparently consists of a positively charged amino acid followed by a number of hydrophobic residues, a well-known endoplasmic reticulum localization signal (Fig. S6) [57]). The mitochondrion transit peptides are enriched in arginine and leucine. For other subcellular (Figs. S5 and S6) and suborganellar (Figs. S9-S15classes of proteins, we also found high attention regions near both N- and C-termini. Although most of them appear to be caused by terminus bias, several known signals were recognized; in particular, a well-known N-terminal endoplasmic reticulum signal peptide recognized in extracellular proteins (Fig. S4) was also seen in proteins from cytoplasmic granule (Fig. 4A) and cell surface (Fig. 4B), both of which pass through the endoplasmic reticulum on the way to those localizations [57]. This N-terminal signal was particularly strong in proteins belonging to the endoplasmic reticulum lumen (Fig. 4C), where it was seen together with a C-terminal KDEL/HDEL signal (Fig. 4E), which is an endoplasmic reticulum retention signal [58], [59], [60]. We also found a clear C-terminal SRL/SKL/SRM signal in peroxisome proteins (Fig. 4F), which is a targeting signal for proteins belonging to the peroxisome [52], [53].

To analyze the attention in the middle of proteins, we used the GLAM2 tool in the MEME suite, and we demonstrate the effectiveness of this approach by visualizing the attention of proteins located in the nucleus (Fig. 5). We also found a clear L/I/VxxxxxxL/V/I/F motif in proteins belonging to the cytoplasm (Fig. S16), which is known to be a nuclear export signal [54]. Note that the analysis by GLAM2 was influenced by the number of top amino acids and the window size we chose. The parameters of the GLAM2 itself could also affect the results. Changing the parameter configuration would result in different visualization results, but the general motif information remains the same. For other localizations, where the mechanisms are unclear, we provide a list of attention visualizations in Figs. S16-S21. They could be used as candidate motifs for these localizations.

There are limitations to the MULocDeep method, which may lead to several future studies. First of all, more advanced machine-learning methods, such as graph-based neural networks, could be applied for feature representation and localization prediction. Secondly, a more rigorous confidence assessment of each predicted localization could be provided instead of the current prediction probabilities, and each predicted motif can be given a confidence assessment as well. Furthermore, the prediction performance could be further improved by building species-specific models. Finally, future applications could be extended to localization-related disease studies, e.g., to predict the impact of a mutation in localization. With that said, we are still a long way from understanding protein localization fully. Given the complexities of decoding signal peptides or extracting features that can distinguish detailed levels of localizations, the interpretability of MUlocDeep makes it possible to generate hypotheses regarding protein sorting mechanisms. Some of them can be verified by current knowledge, making the rest worth exploring by combining computational prediction methods and experimental verification. By making the datasets, the software code, and the MULocDeep website publicly accessible, our study constitutes a major step toward understanding the protein localization mechanism.

## 5 Availability

The mass spectrometry proteomics data have been deposited to the ProteomeXchange Consortium via the PRIDE [61] partner repository with the dataset identifier PXD019987. Datasets used to train and test the MULocDeep model can be found in the GitHub repository (https://github.com/yuexujiang/MULocDeep). All the codes for training and testing the MULocDeep model can be found in the GitHub repository (https://github.com/yuexujiang/MULocDeep).

## Funding

This work was supported by the US National Institutes of Health grants R21-LM012790 and R35-GM126985.

## 7 Contributions

D.X. conceived and supervised the project. Y.J. designed the neural network, developed the code, wrote most of the manuscript. D.W. helped with the neural network design, prepared the UniProt dataset. H.E., P.K., and I.M.M. prepared the mitochondrial proteome dataset, provided biological insights, and helped write the manuscript. D.W. and Y.Y. helped with the result evaluation and comparison. Y.Y. developed the MULocDeep website.

## CRediT authorship contribution statement

**Yuexu Jiang:** Conceptualization, Methodology, Software, Validation, Data Curation, Writing - original draft, Visualization. **Duolin Wang**: Software, Validation, Writing - review & editing. **Yifu Yao:** Software, Visualization. **Holger Eubel:** Investigation, Writing - review & editing, Resources. **Patrick Künzler:** Investigation, Writing - review & editing, Resources. **Ian Max Møller:** Investigation, Writing - review & editing, Resources. **Dong Xu:** Resources, Writing - review & editing, Supervision, Project administration, Funding acquisition.

## Declaration of Competing Interest

The authors declare that they have no known competing financial interests or personal relationships that could have appeared to influence the work reported in this paper.

## Appendix A Supplementary data

Supplementary data 1 Identified Vicia Proteins (as Medicago IDs) with Medicago Sequences and Arabidopsis Match

Supplementary data 2 Identified Solanum Proteins with Sequences and Arabidopsis Match

Supplementary data 3 Identified Arabidopsis Proteins with Sequences
