# Supplementary material for: MULocDeep: A deep-learning framework for protein subcellular and suborganellar localization prediction with residue-level interpretation
Source: Comput Struct Biotechnol J. 2021 Aug 18;19:4825–39. doi: 10.1016/j.csbj.2021.08.027 (PMC8426535; doi:10.1016/j.csbj.2021.08.027)
Supplement: Supplementary data 4 [file mmc4.docx]

| **Table S1**. The statistics of samples in each localization in the *UniLoc* dataset | | | | | | | |
| --- | --- | --- | --- | --- | --- | --- | --- |
| Subcellular localization | No. of samples in testing dataset | No. of samples in non-redundant training dataset | No. of samples in redundant training dataset | Suborganellar localization | No. of samples in testing dataset | No. of samples in non-redundant training dataset | No. of samples in redundant training dataset |
| Nucleus | 1136 | 6650 | 19974 | Nucleolus | 109 | 433 | 1938 |
|  |  |  |  | Nucleoplasm | 49 | 258 | 891 |
|  |  |  |  | Membrane | 61 | 241 | 1134 |
|  |  |  |  | Nucleus matrix | 10 | 41 | 194 |
|  |  |  |  | Nucleus speckle | 38 | 241 | 764 |
|  |  |  |  | PML body | 9 | 78 | 249 |
|  |  |  |  | Cajal body | 6 | 25 | 125 |
|  |  |  |  | Chromosome | 126 | 597 | 2703 |
| Cytoplasm | 984 | 3723 | 16038 | Vesicle | 105 | 677 | 2505 |
|  |  |  |  | Cytoskeleton | 245 | 1490 | 5760 |
|  |  |  |  | Myofibril | 19 | 139 | 318 |
|  |  |  |  | Cytosol | 126 | 534 | 2418 |
|  |  |  |  | Perinuclear region | 50 | 349 | 1101 |
|  |  |  |  | Cell cortex | 27 | 199 | 426 |
|  |  |  |  | Cytoplasmic granule | 18 | 116 | 329 |
|  |  |  |  | P-body | 15 | 67 | 266 |
| Sereted | 441 | 1826 | 11018 | Extracellular space | 40 | 611 | 1379 |
| Mitochondrion | 183 | 784 | 4193 | Inner membrane | 73 | 418 | 1883 |
|  |  |  |  | Intermembrane space | 16 | 25 | 335 |
|  |  |  |  | Outer membrane | 32 | 160 | 714 |
|  |  |  |  | Mitochondrion matrix | 42 | 187 | 1065 |
|  |  |  |  | Membrane | 21 | 107 | 438 |
| Membrane | 101 | 2645 | 8582 | Clathrin-coated pit | 4 | 67 | 147 |
|  |  |  |  | Coated pit | 1 | 36 | 101 |
|  |  |  |  | Membrane raft | 13 | 88 | 279 |
|  |  |  |  | Caveola | 4 | 43 | 153 |
|  |  |  |  | Cell membrane | 590 | 4839 | 13379 |
|  |  |  |  | Cell surface | 11 | 103 | 235 |
| Endoplasmic | 62 | 278 | 981 | ER lumen | 15 | 166 | 409 |
|  |  |  |  | ER membrane | 198 | 1211 | 4630 |
|  |  |  |  | ER-Golgi intermediate compartment | 11 | 64 | 266 |
|  |  |  |  | Microsome | 23 | 336 | 823 |
|  |  |  |  | Sarcoplasmic reticulum | 6 | 57 | 163 |
| Plastid | 2 | 5 | 103 | Amyloplast | 3 | 1 | 66 |
|  |  |  |  | Chloroplast membrane | 24 | 85 | 393 |
|  |  |  |  | Chloroplast stroma | 27 | 43 | 334 |
|  |  |  |  | Chloroplast thylakoid lumen | 6 | 9 | 59 |
|  |  |  |  | Chloroplast thylakoid membrane | 29 | 41 | 680 |
| Golgi apparatus | 46 | 263 | 1045 | Trans-Golgi network | 35 | 202 | 744 |
|  |  |  |  | Cis-Golgi network | 11 | 44 | 248 |
|  |  |  |  | Membrane | 88 | 484 | 2275 |
|  |  |  |  | Golgi stack membrane | 9 | 58 | 301 |
| Lysosome | 23 | 203 | 1063 | Membrane | 34 | 152 | 724 |
| Peroxisome | 29 | 168 | 678 | Membrane | 13 | 90 | 269 |

| **Table S2**. Statistics from the Datasets Used in the DeepMito Method | | |
| --- | --- | --- |
| Compartment | Datasets | |
|  | SM424-18 | SubMitoPred |
| Outer membrane | 74 | 82 |
| Inner membrane | 190 | 282 |
| Intermembrane space | 25 | 32 |
| Matrix | 135 | 174 |
| Total | 424 | 570 |

| **Table S3**. Number of proteins in each localization class in the *DeepLoc* dataset | |
| --- | --- |
| Localization | No. of proteins |
| Nucleus | 4043 |
| Cytoplasm | 2542 |
| Extracellular | 1973 |
| Mitochondrion | 1510 |
| Cell membrane | 1340 |
| Endoplasmic reticulum | 862 |
| Plastid | 757 |
| Golgi apparatus | 356 |
| Lysosome/Vacuole | 321 |
| Peroxisome | 154 |

| **Table S4**. Cross-Validation MCC at the Suborganelle Level with Different Thresholds (0.1-0.9) using *UniLoc-train* dataset | | | | | | | | | | |
| --- | --- | --- | --- | --- | --- | --- | --- | --- | --- | --- |
| Subcellular localization | Suborganellar localization | MCC vs. threshold | | | | | | | | |
|  |  | 0.1 | 0.2 | 0.3 | 0.4 | 0.5 | 0.6 | 0.7 | 0.8 | 0.9 |
| Nucleus | Nucleolus | 0.41 | 0.42 | 0.44 | 0.44 | 0.44 | 0.45 | 0.45 | 0.45 | 0.46 |
|  | Nucleoplasm | 0.23 | 0.24 | 0.25 | 0.25 | 0.25 | 0.24 | 0.23 | 0.22 | 0.2 |
|  | Membrane | 0.2 | 0.22 | 0.22 | 0.23 | 0.24 | 0.25 | 0.26 | 0.27 | 0.29 |
|  | Nucleus matrix | 0.11 | 0.12 | 0.13 | 0.13 | 0.13 | 0.11 | 0.12 | 0.13 | 0.13 |
|  | Nucleus speckle | 0.31 | 0.31 | 0.31 | 0.32 | 0.32 | 0.32 | 0.32 | 0.32 | 0.32 |
|  | PML body | 0.16 | 0.15 | 0.12 | 0.11 | 0.08 | 0.09 | 0.1 | 0.12 | 0.13 |
|  | Cajal body | 0.05 | 0.03 | 0.02 | 0.02 | 0.02 | 0.02 | 0.01 | 0.01 | 0 |
|  | Chromosome | 0.46 | 0.47 | 0.47 | 0.48 | 0.49 | 0.49 | 0.49 | 0.5 | 0.5 |
| Cytoplasm | Vesicle | 0.21 | 0.21 | 0.22 | 0.23 | 0.23 | 0.23 | 0.24 | 0.24 | 0.24 |
|  | Cytoskeleton | 0.5 | 0.5 | 0.51 | 0.51 | 0.52 | 0.52 | 0.52 | 0.52 | 0.53 |
|  | Myofibril | 0.21 | 0.24 | 0.22 | 0.2 | 0.17 | 0.15 | 0.12 | 0.1 | 0.09 |
|  | Cytosol | 0.29 | 0.29 | 0.29 | 0.29 | 0.29 | 0.3 | 0.3 | 0.3 | 0.3 |
|  | Perinuclear region | 0.12 | 0.11 | 0.11 | 0.11 | 0.11 | 0.11 | 0.11 | 0.11 | 0.09 |
|  | Cell cortex | 0.12 | 0.13 | 0.13 | 0.14 | 0.14 | 0.13 | 0.13 | 0.12 | 0.12 |
|  | Cytoplasmic granule | 0.11 | 0.11 | 0.13 | 0.11 | 0.11 | 0.13 | 0.13 | 0.14 | 0.15 |
|  | P-body | 0.16 | 0.17 | 0.18 | 0.19 | 0.19 | 0.18 | 0.19 | 0.18 | 0.19 |
| Secreted | Extracellular space | 0.67 | 0.68 | 0.69 | 0.69 | 0.69 | 0.69 | 0.7 | 0.7 | 0.7 |
| Mitochondrion | Inner membrane | 0.63 | 0.63 | 0.64 | 0.64 | 0.64 | 0.64 | 0.65 | 0.65 | 0.65 |
|  | Intermembrane space | 0.31 | 0.34 | 0.34 | 0.36 | 0.36 | 0.37 | 0.37 | 0.36 | 0.37 |
|  | Outer membrane | 0.39 | 0.41 | 0.43 | 0.44 | 0.45 | 0.47 | 0.47 | 0.48 | 0.48 |
|  | Mitochondrion matrix | 0.55 | 0.56 | 0.57 | 0.58 | 0.58 | 0.57 | 0.57 | 0.57 | 0.58 |
|  | Membrane | 0.13 | 0.12 | 0.13 | 0.13 | 0.14 | 0.15 | 0.16 | 0.17 | 0.15 |
| Membrane | Clathrin-coated pit | 0.18 | 0.2 | 0.22 | 0.22 | 0.23 | 0.24 | 0.25 | 0.25 | 0.24 |
|  | Coated pit | 0.17 | 0.18 | 0.19 | 0.2 | 0.22 | 0.22 | 0.21 | 0.17 | 0.17 |
|  | Membrane raft | 0.08 | 0.06 | 0.06 | 0.04 | 0.04 | 0.04 | 0.04 | 0.02 | 0.01 |
|  | Caveola | 0.16 | 0.18 | 0.15 | 0.17 | 0.18 | 0.17 | 0.14 | 0.13 | 0.13 |
|  | Cell membrane | 0.68 | 0.68 | 0.69 | 0.69 | 0.69 | 0.69 | 0.69 | 0.69 | 0.69 |
|  | Cell surface | 0.1 | 0.1 | 0.1 | 0.1 | 0.1 | 0.1 | 0.1 | 0.09 | 0.08 |
| Endoplasmic | ER lumen | 0.69 | 0.71 | 0.73 | 0.72 | 0.73 | 0.73 | 0.74 | 0.74 | 0.73 |
|  | ER membrane | 0.6 | 0.6 | 0.61 | 0.61 | 0.61 | 0.61 | 0.61 | 0.61 | 0.61 |
|  | ER-Golgi intermediate compartment | 0.26 | 0.29 | 0.29 | 0.31 | 0.32 | 0.32 | 0.3 | 0.27 | 0.21 |
|  | Microsome | 0.47 | 0.49 | 0.5 | 0.51 | 0.51 | 0.51 | 0.51 | 0.51 | 0.48 |
|  | Sarcoplasmic reticulum | 0.17 | 0.21 | 0.21 | 0.23 | 0.25 | 0.24 | 0.23 | 0.23 | 0.23 |
| Plastid | Amyloplast | 0.31 | 0.34 | 0.35 | 0.37 | 0.36 | 0.31 | 0.33 | 0.34 | 0.33 |
|  | Chloroplast membrane | 0.44 | 0.44 | 0.45 | 0.45 | 0.45 | 0.46 | 0.47 | 0.47 | 0.48 |
|  | Chloroplast stroma | 0.54 | 0.54 | 0.54 | 0.55 | 0.55 | 0.54 | 0.55 | 0.54 | 0.55 |
|  | Chloroplast thylakoid lumen | 0.33 | 0.33 | 0.35 | 0.33 | 0.32 | 0.34 | 0.33 | 0.29 | 0.29 |
|  | Chloroplast thylakoid membrane | 0.54 | 0.55 | 0.56 | 0.56 | 0.56 | 0.56 | 0.56 | 0.56 | 0.56 |
| Golgi apparatus | Trans-Golgi network | 0.16 | 0.16 | 0.15 | 0.15 | 0.14 | 0.13 | 0.12 | 0.1 | 0.09 |
|  | Cis-Golgi network | 0.24 | 0.28 | 0.28 | 0.28 | 0.29 | 0.29 | 0.28 | 0.25 | 0.24 |
|  | Membrane | 0.5 | 0.51 | 0.51 | 0.52 | 0.52 | 0.52 | 0.51 | 0.51 | 0.51 |
|  | Golgi stack membrane | 0.34 | 0.34 | 0.35 | 0.36 | 0.35 | 0.37 | 0.36 | 0.38 | 0.37 |
| Lysosome | Membrane | 0.28 | 0.28 | 0.28 | 0.28 | 0.28 | 0.28 | 0.27 | 0.27 | 0.27 |
| Peroxisome | Membrane | 0.52 | 0.53 | 0.52 | 0.51 | 0.52 | 0.52 | 0.53 | 0.53 | 0.53 |
|  | | | | | | | | | | |

| **Table S5**. Searching Range and Optimized Values of the Hyperparameters Through the Bayesian Optimization Process | | | | | | | | | |
| --- | --- | --- | --- | --- | --- | --- | --- | --- | --- |
| Parameters | Range | Optimized Values | | | | | | | |
|  |  | **M1** | M2 | M3 | M4 | M5 | M6 | M7 | M8 |
| Hidden dimension in LSTM | [32, 490] | 180 | 220 | 389 | 303 | 264 | 392 | 230 | 273 |
| Hidden dimension in attention | [32, 430] | 369 | 189 | 194 | 401 | 302 | 350 | 275 | 420 |
| Number of heads in attention | [16, 64] | 41 | 27 | 47 | 37 | 38 | 38 | 57 | 26 |
| Regularizer of L2 loss in attention | [10E-5, 10E-3] | 10E-5 | 10E-5 | 3E-4 | 0.001 | 10E-5 | 0.001 | 2.7E-4 | 10E-5 |
| Regularizer of penalization in attention | [10E-5, 10E-3] | 7.2E-4 | 10E-5 | 1.3E-4 | 10E-5 | 0.001 | 0.001 | 10E-5 | 0.001 |
| Dropout rate of input layer | [0.1, 0.75] | 0.1 | 0.1 | 0.116 | 0.1 | 0.1 | 0.1 | 0.1 | 0.1 |
| Dropout rate of hidden layers | [0.1, 0.75] | 0.1 | 0.1 | 0.133 | 0.1 | 0.1 | 0.1 | 0.1 | 0.1 |
| Performance (accuracy) | | 0.789 | 0.755 | 0.769 | 0.761 | 0.780 | 0.761 | 0.751 | 0.772 |
| Each sub-model (M1-M8) was optimized individually. The hyperparameters of M1, which achieved the highest prediction accuracy, were selected as the final optimum hyperparameters. | | | | | | | | | |

| **Table S6**. Performance of Ensemble Models on Different Hyperparameter Configurations | | | | | | |
| --- | --- | --- | --- | --- | --- | --- |
| Hyperparameters that each sub-model uses | | Config 1 | Config 2 | Config 3 | Config 4 | Config 5 |
| Hyperparameters | Hidden dimension in LSTM | See Table S1 | 220 | 389 | 256 | 180 |
|  | Hidden dimension in attention | See Table S1 | 189 | 194 | 256 | 369 |
|  | Number of heads in attention | See Table S1 | 27 | 47 | 30 | 41 |
|  | Regularizer of L2 loss in attention | See Table S1 | 10E-5 | 3E-4 | 0.001 | 10E-5 |
|  | Regularizer of penalization in attention | See Table S1 | 10E-5 | 1.3E-4 | 0.001 | 7.2E-4 |
|  | Dropout rate of input layer | See Table S1 | 0.1 | 0.116 | 0.2 | 0.1 |
|  | Dropout rate of hidden layers | See Table S1 | 0.1 | 0.133 | 0.5 | 0.1 |
| Performance | Accuracy | 0.759 | 0.753 | 0.765 | 0.743 | 0.761 |
|  | Gorodkin | 0.707 | 0.701 | 0.715 | 0.688 | 0.711 |
|  | MCC-Nucleus | 0.733 | 0.722 | 0.739 | 0.716 | 0.725 |
|  | MCC-Cytoplasm | 0.550 | 0.538 | 0.563 | 0.536 | 0.549 |
|  | MCC-Extracellular | 0.894 | 0.896 | 0.897 | 0.887 | 0.896 |
|  | MCC-Mitochondrion | 0.811 | 0.832 | 0.832 | 0.783 | 0.823 |
|  | MCC-Cell membrane | 0.695 | 0.677 | 0.683 | 0.679 | 0.696 |
|  | MCC-ER | 0.568 | 0.571 | 0.589 | 0.543 | 0.602 |
|  | MCC-Plastid | 0.908 | 0.919 | 0.896 | 0.868 | 0.901 |
|  | MCC-Glogi apparatus | 0.449 | 0.455 | 0.535 | 0.449 | 0.464 |
|  | MCC-Lysosome | 0.149 | 0.096 | 0.157 | 0.062 | 0.208 |
|  | MCC-Peroxisome | 0.449 | 0.412 | 0.423 | 0.370 | 0.412 |
| Config 1: Each sub-model uses its own optimized hyperparameters. | | | | | | |
| Config 2: All sub-models use the same but not the final optimum hyperparameters, in this case, the hyperparameters in sub-model 2. | | | | | | |
| Config 3: All sub-models use the same but not the final optimum hyperparameters, in this case, the hyperparameters in sub-model 3. | | | | | | |
| Config 4: All sub-models use the same random hyperparameters. | | | | | | |
| Config 5: All sub-models use the same final optimum hyperparameters.  Performance is measured by accuracy, Gorodkin value, and Matthew’s correlation coefficient (MCC). | | | | | | |

| **Table S7**. Performance of Single Models on Different Hyperparameter Configurations | | | | | | | | | |
| --- | --- | --- | --- | --- | --- | --- | --- | --- | --- |
|  | | M1 | M2 | M3 | M4 | M5 | M6 | M7 | M8 |
| Performance | Accuracy | 0.735 | 0.724 | 0.716 | 0.726 | 0.729 | 0.729 | 0.730 | 0.717 |
|  | Gorodkin | 0.679 | 0.666 | 0.656 | 0.669 | 0.672 | 0.673 | 0.673 | 0.657 |
|  | MCC-Nucleus | 0.700 | 0.674 | 0.683 | 0.709 | 0.692 | 0.715 | 0.685 | 0.682 |
|  | MCC-Cytoplasm | 0.528 | 0.466 | 0.468 | 0.505 | 0.507 | 0.505 | 0.490 | 0.482 |
|  | MCC-Extracellular | 0.879 | 0.867 | 0.879 | 0.869 | 0.879 | 0.874 | 0.877 | 0.870 |
|  | MCC-Mitochondrion | 0.770 | 0.802 | 0.801 | 0.749 | 0.783 | 0.767 | 0.779 | 0.777 |
|  | MCC-Cell membrane | 0.678 | 0.688 | 0.621 | 0.679 | 0.678 | 0.654 | 0.686 | 0.640 |
|  | MCC-ER | 0.544 | 0.547 | 0.540 | 0.505 | 0.505 | 0.524 | 0.562 | 0.522 |
|  | MCC-Plastid | 0.874 | 0.874 | 0.808 | 0.871 | 0.858 | 0.883 | 0.868 | 0.845 |
|  | MCC-Glogi apparatus | 0.323 | 0.316 | 0.389 | 0.366 | 0.361 | 0.306 | 0.394 | 0.329 |
|  | MCC-Lysosome | 0.095 | 0.137 | 0.144 | 0.173 | 0.112 | 0.166 | 0.111 | 0.077 |
|  | MCC-Peroxisome | 0.400 | 0.324 | 0.300 | 0.189 | 0.342 | 0.452 | 0.295 | 0.481 |
| Each sub-model (M1-M8) used the same final optimum hyperparameters. | | | | | | | | | |


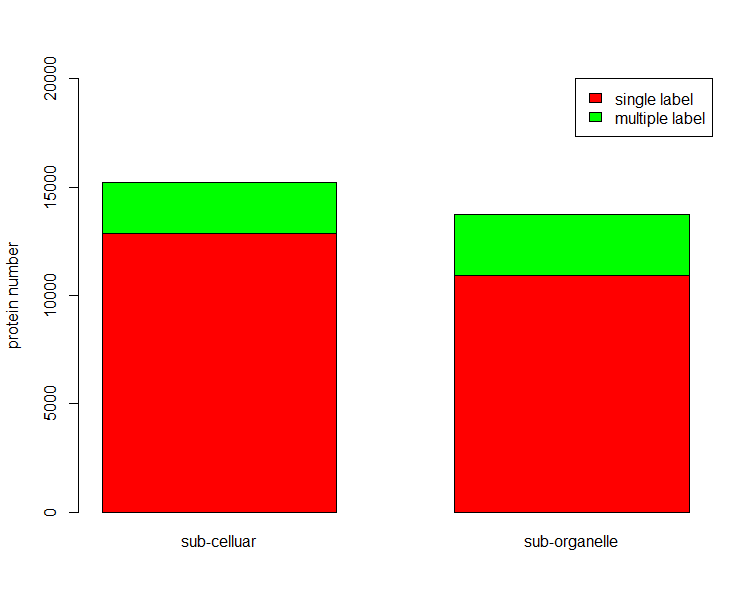


**Figure S1**. Distribution of eukaryotic proteins collected from the UniProt database with experimental evidence at the transcript or protein level. Proteins with multiple localization labels may be at both the subcellular and suborganelle level. All the localization annotations have experimental evidence (ECO:0000269). The Uniprot release is 2020_04.


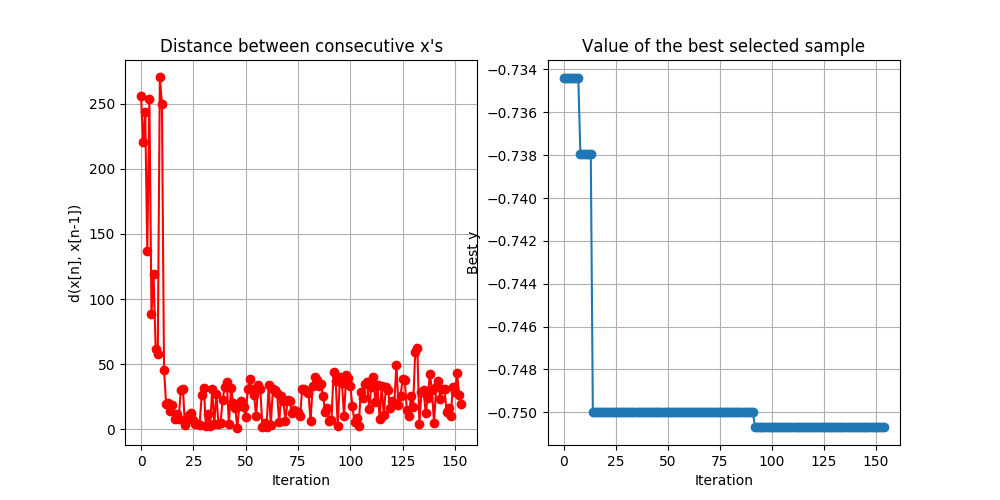


**Figure S2**. Bayesian optimization process.


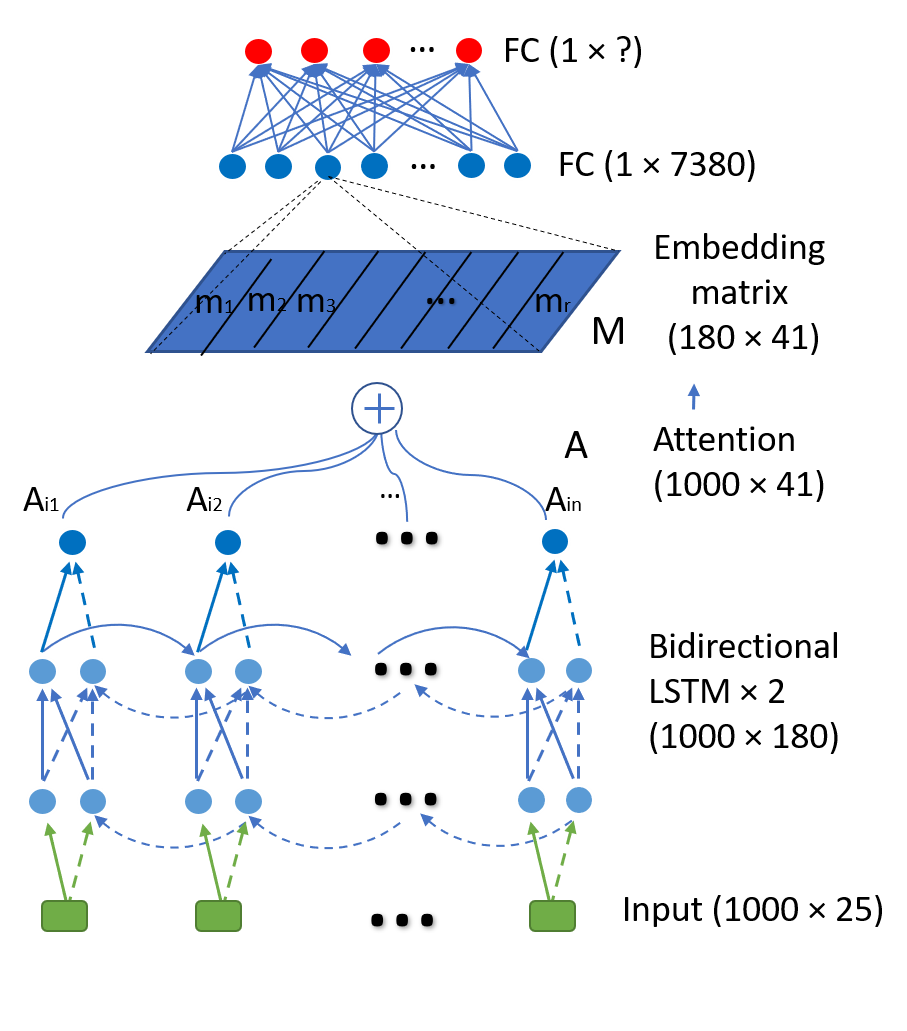


**Figure S3**. Deep learning architecture of the variant model.


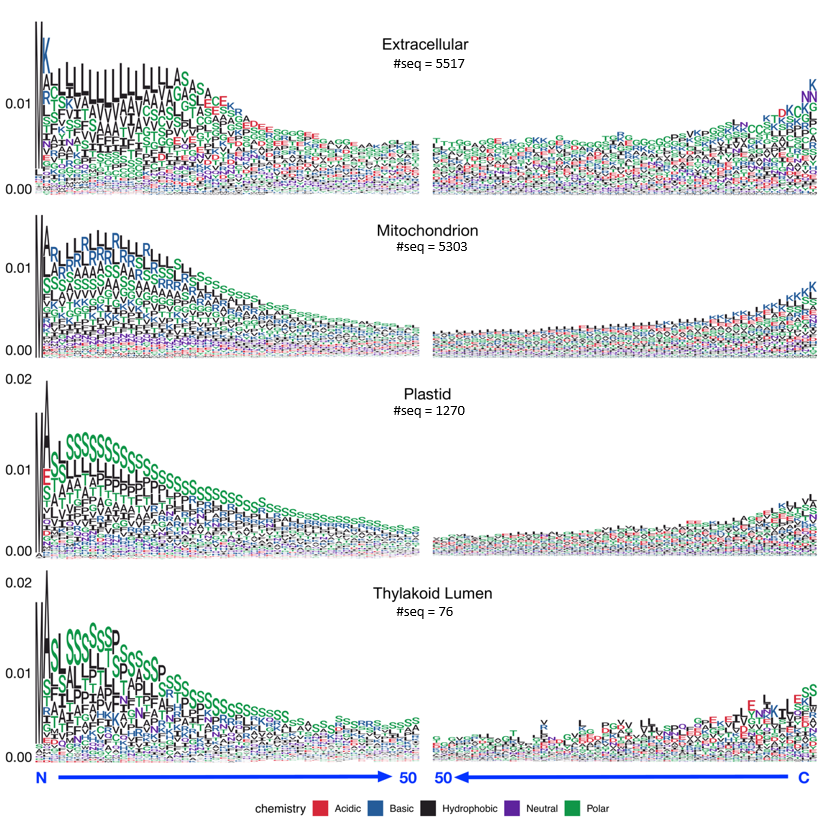


**Figure S4**. The attention weights at the N-terminus (left column) and the C-terminus (right column) for proteins localized in the extracellular, mitochondrion, plastid, and thylakoid lumen.


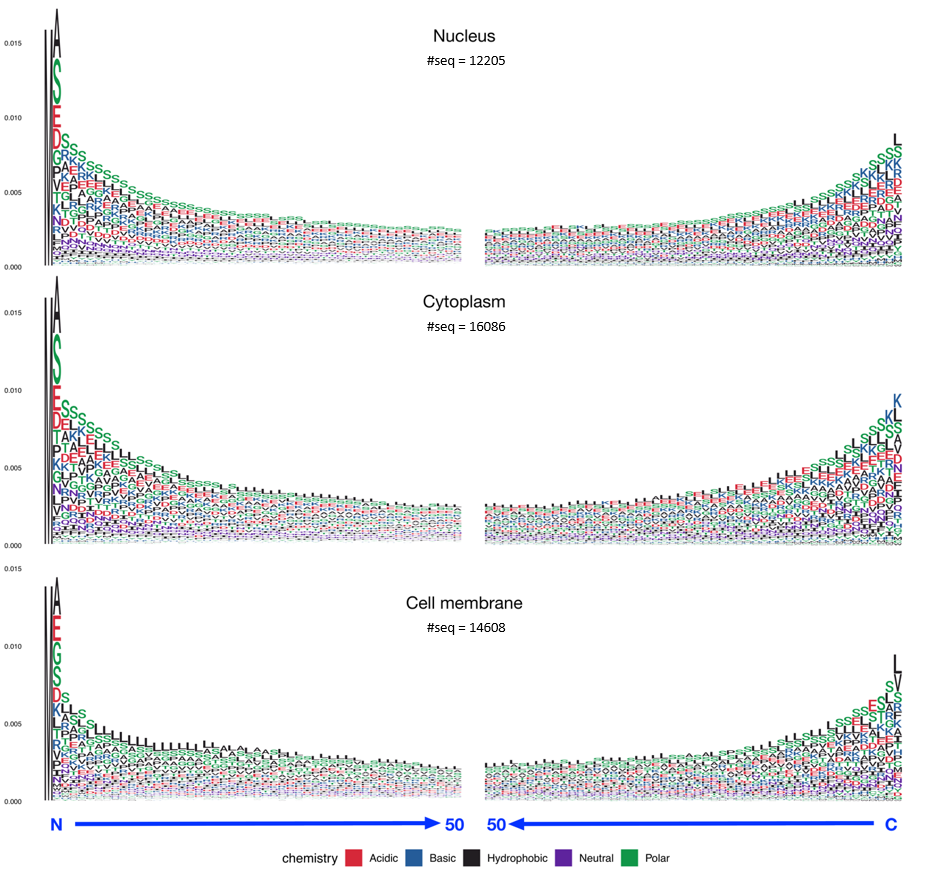


**Figure S5**. The attention weights at the N-terminus (left column) and the C-terminus (right column) for proteins localized at the nucleus, cytoplasm, and cell membrane.


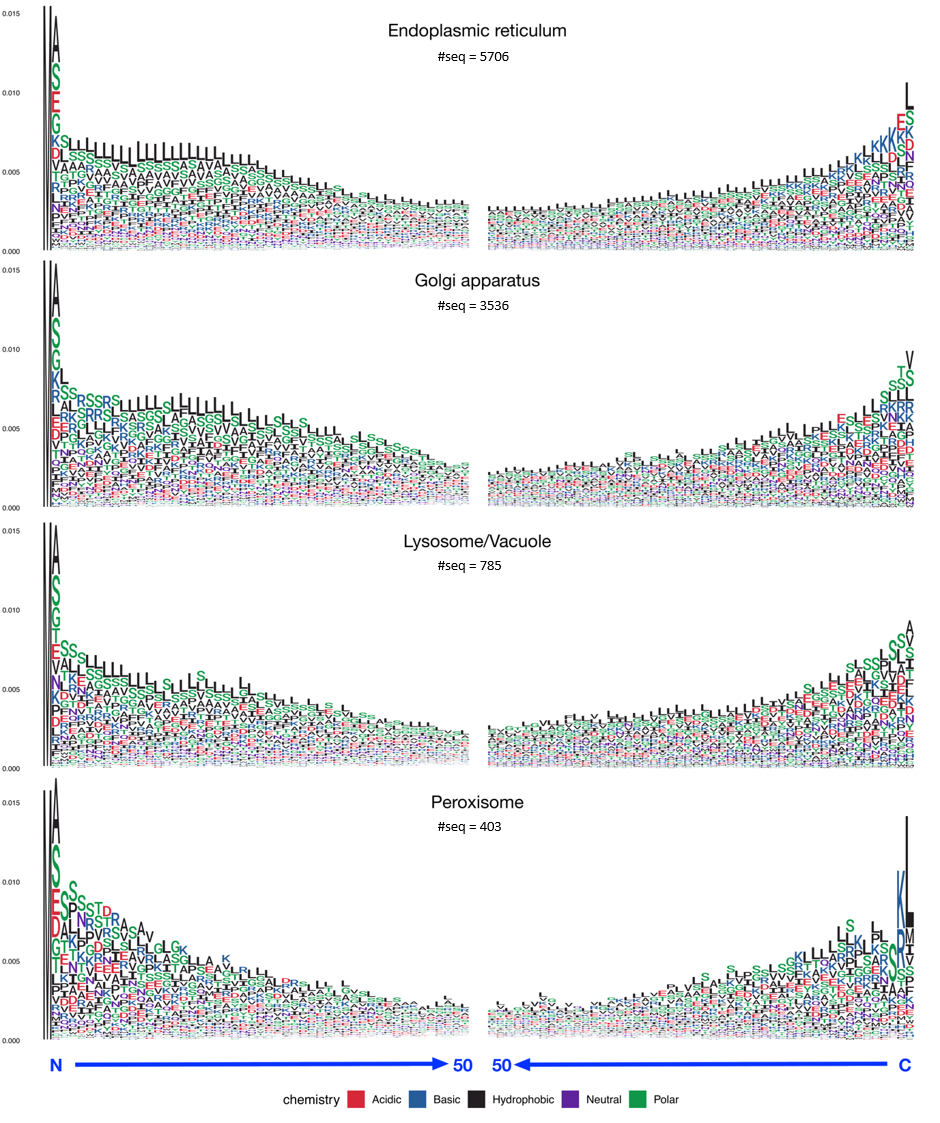


**Figure S6**. The attention weights at the N-terminus (left column) and the C-terminus (right column) for proteins localized at the endoplasmic reticulum, Golgi apparatus, lysosome, and peroxisome.


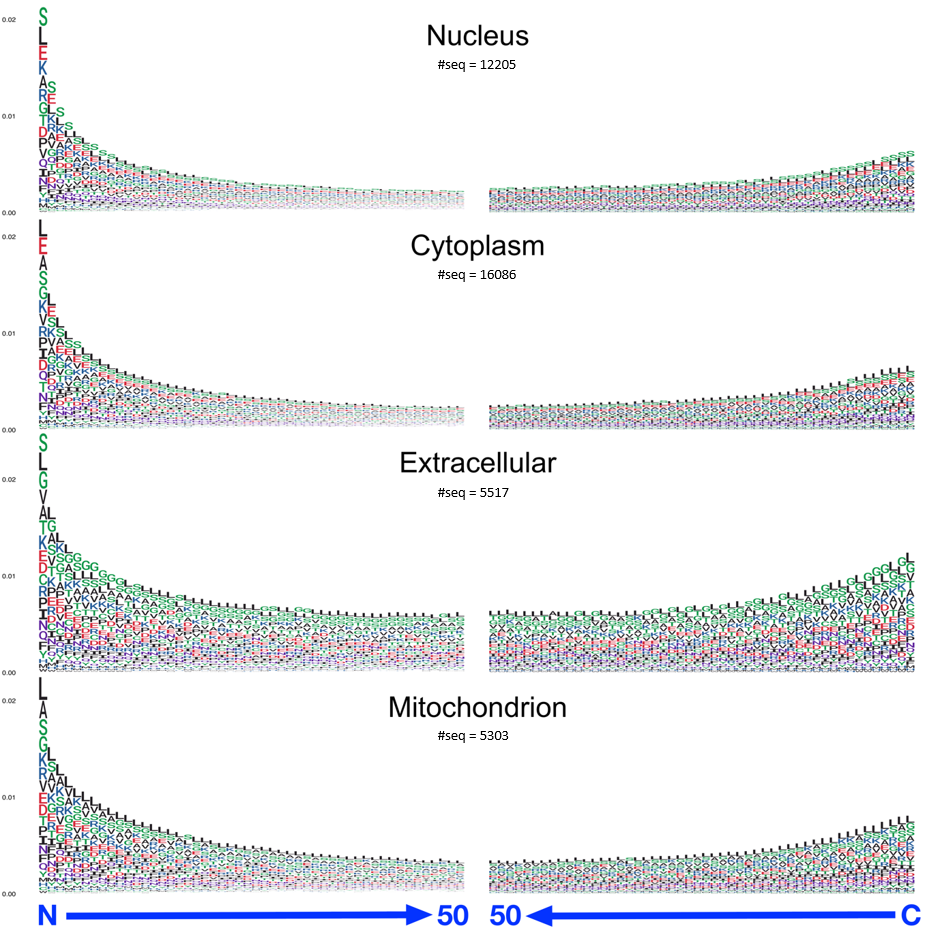


**Figure S7**. The attention weights at the N-terminus (left column) and the C-terminus (right column) for proteins of randomly shuffled sequences in the nucleus, cytoplasm, extracellular, and mitochondrion.


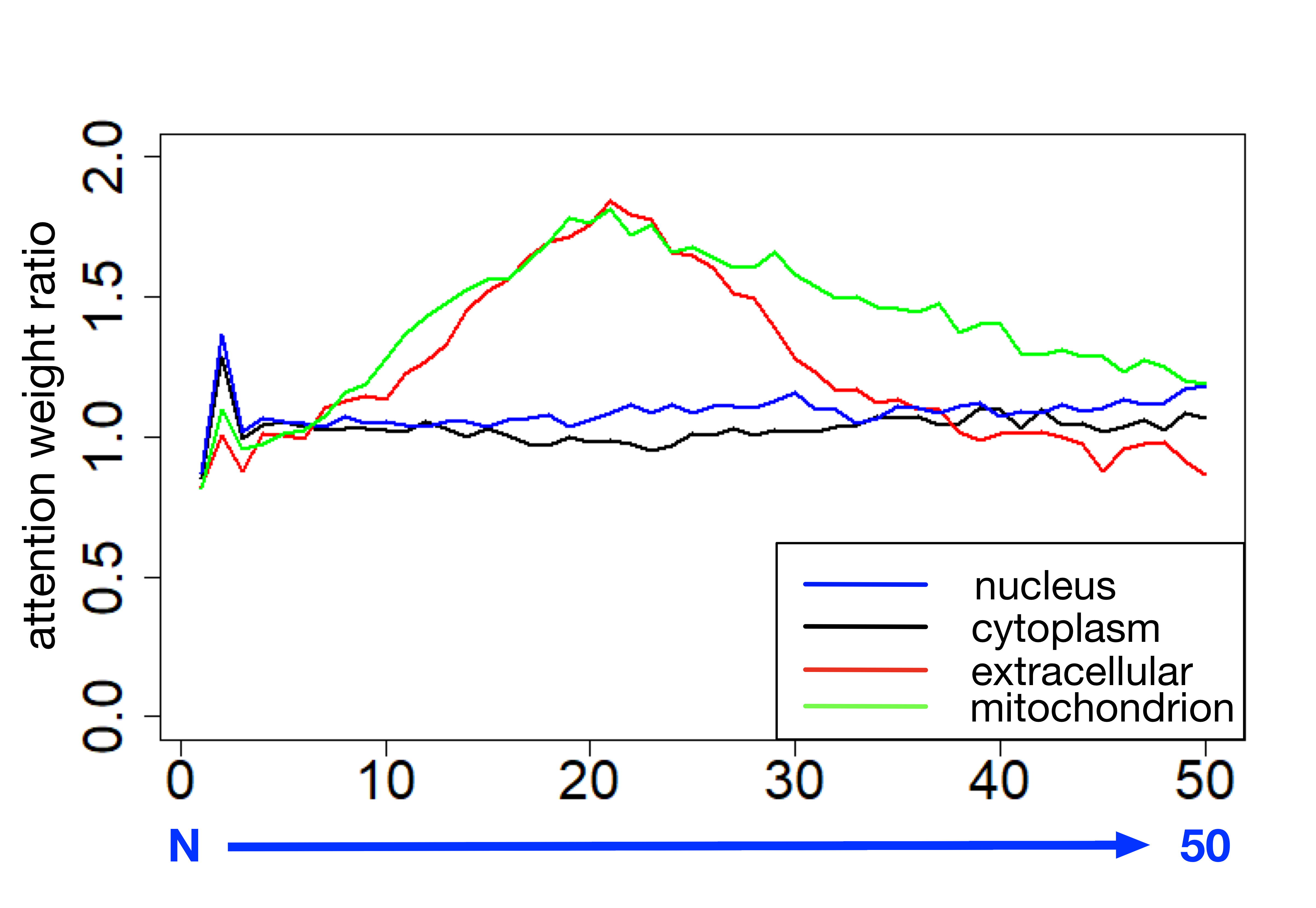


**Figure S8**. The ratios of N-terminal attention weights from real protein sequences to N-terminal attention weights from randomly shuffled protein sequences in the same protein class.


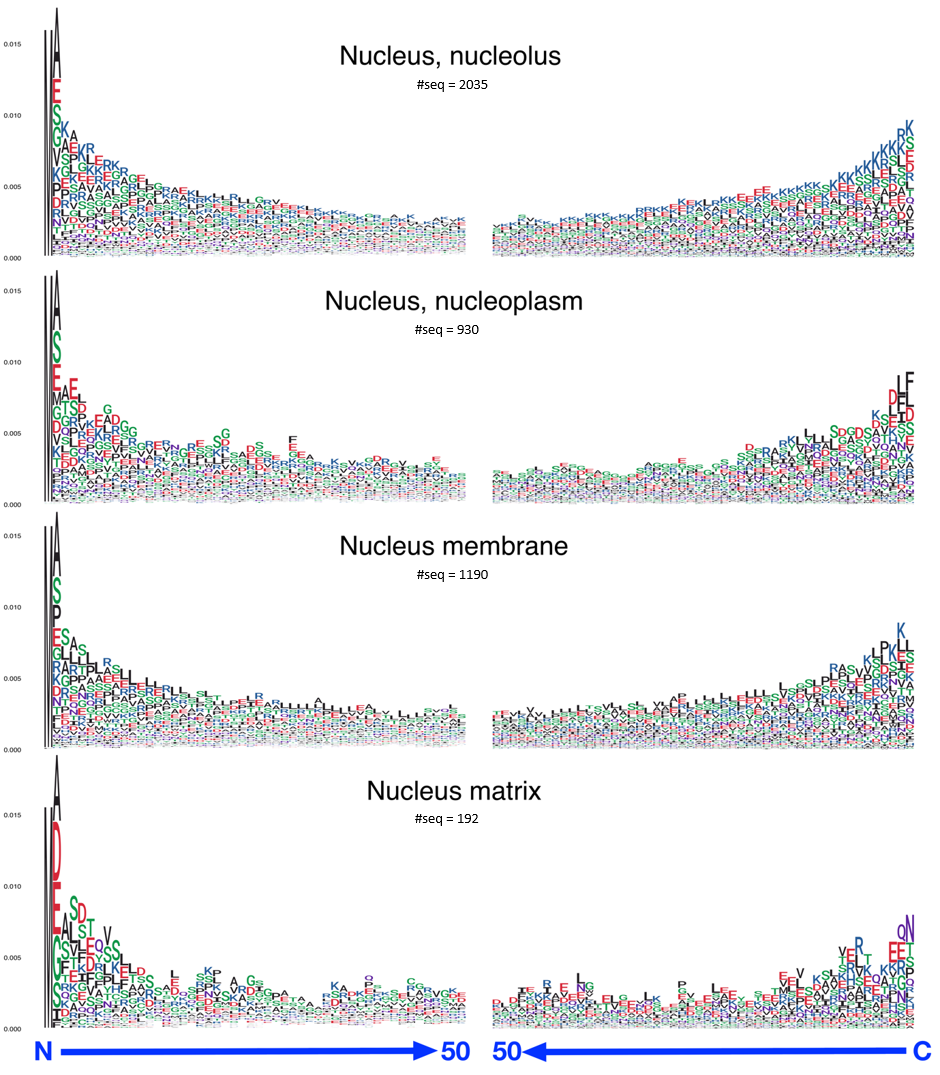


**Figure S9-1**. The attention weights at the N-terminus (left column) and the C-terminus (right column) for protein localizations in the nucleus organelle.


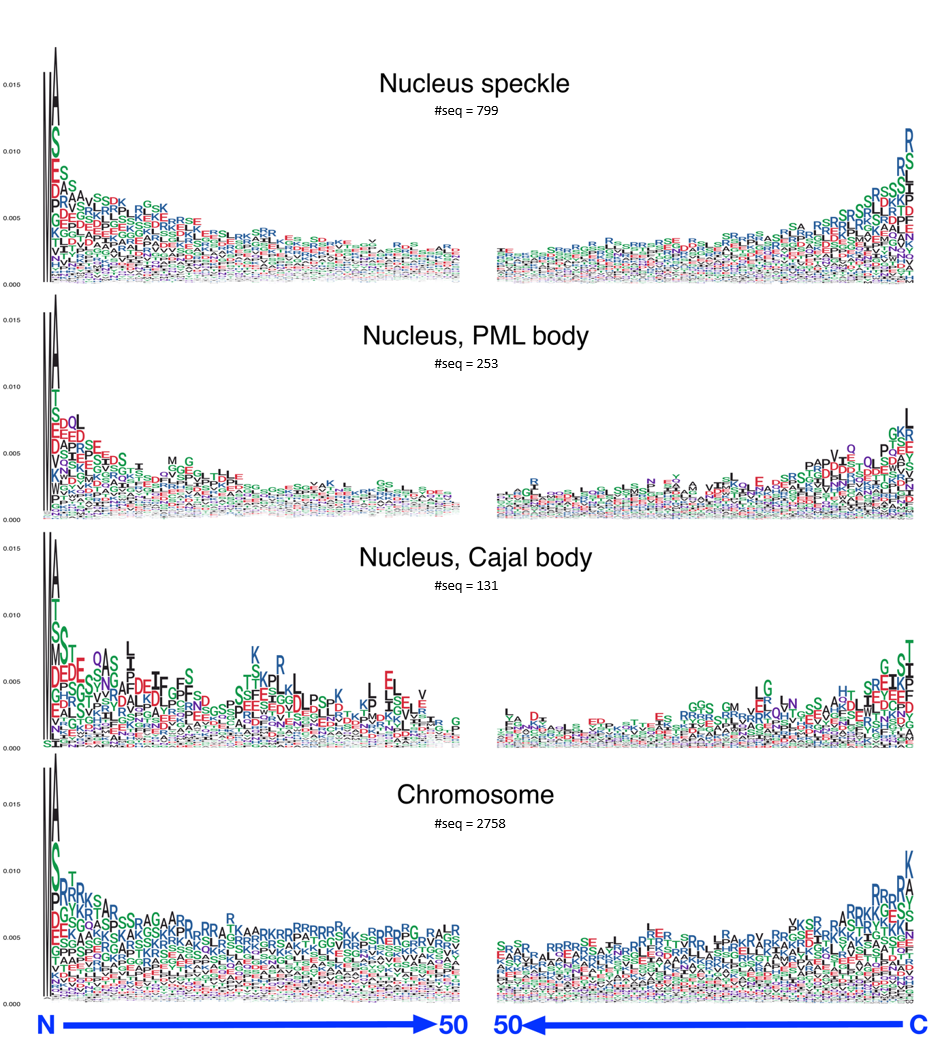


**Figure S9-2**. The attention weights at the N-terminus (left column) and the C-terminus (right column) for protein localizations under the nucleus organelle.


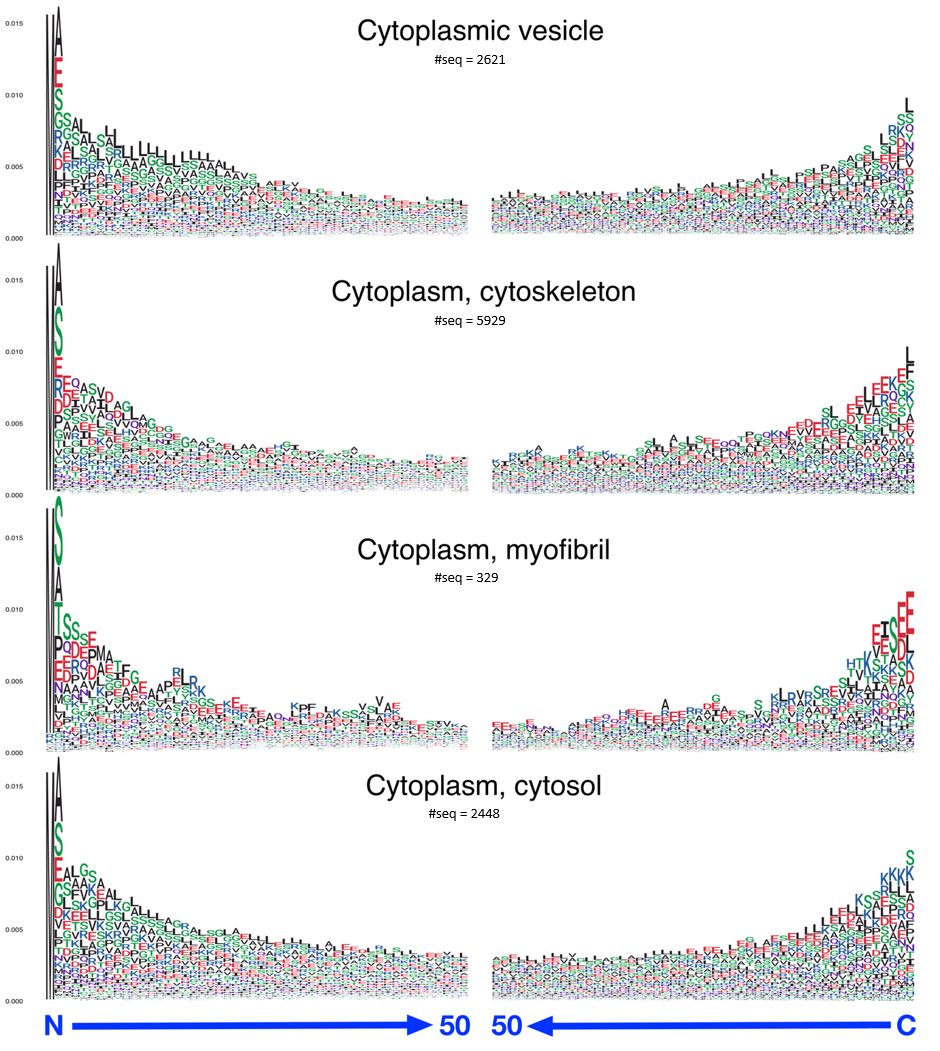


**Figure S10-1**. The attention weights at the N-terminus (left column) and the C-terminus (right column) for protein localizations under the cytoplasm category.


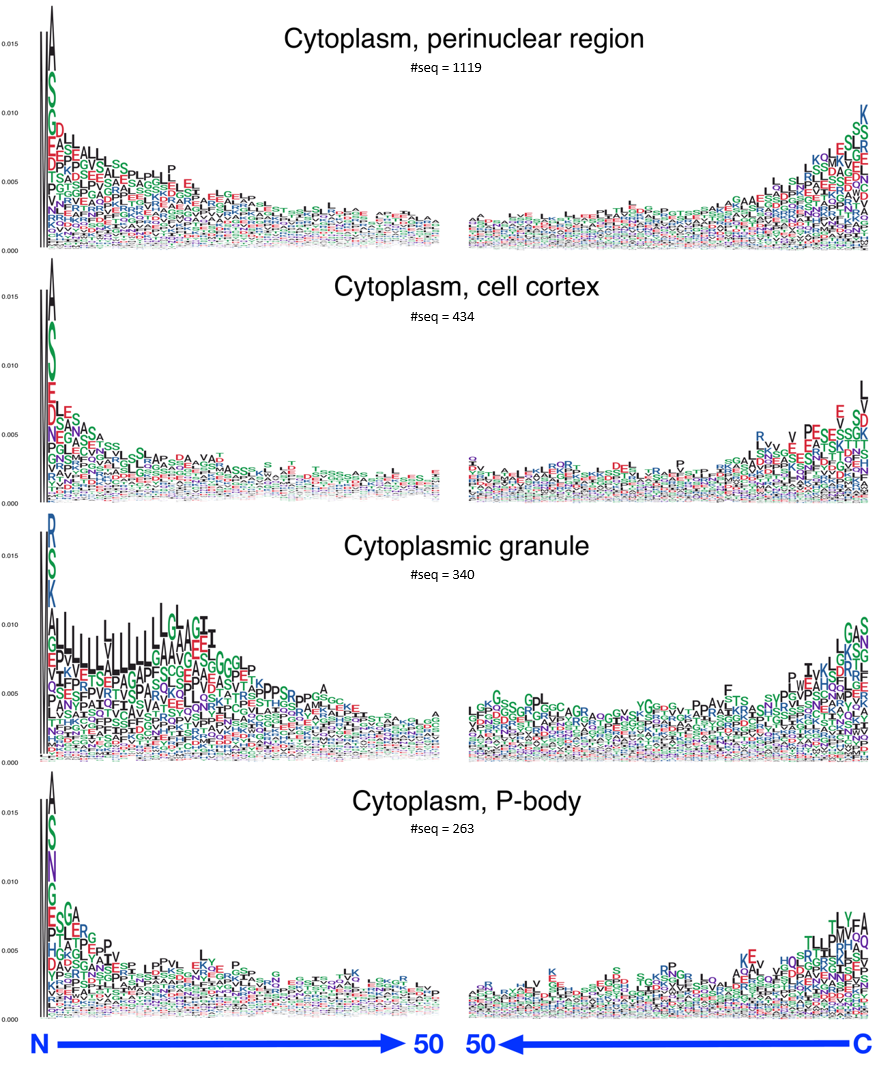


**Figure S10-2**. The attention weights at the N-terminus (left column) and the C-terminus (right column) for protein localizations under the cytoplasm category.


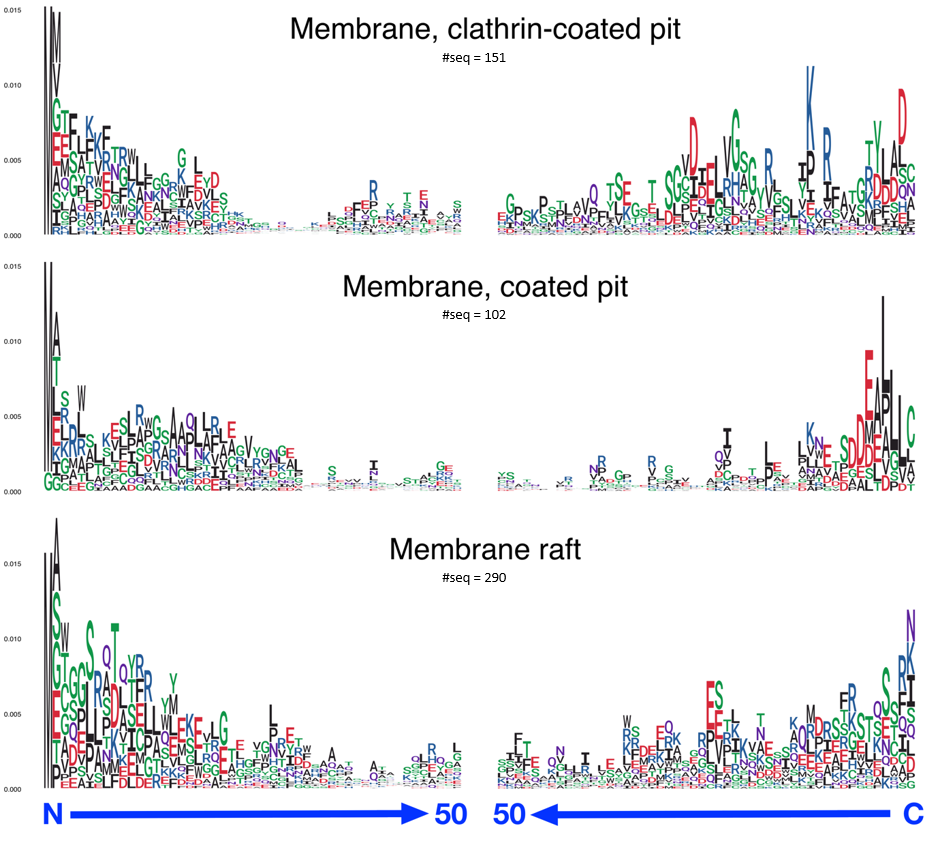


**Figure S11-1**. The attention weights at the N-terminus (left column) and the C-terminus (right column) for protein localizations under the membrane category.


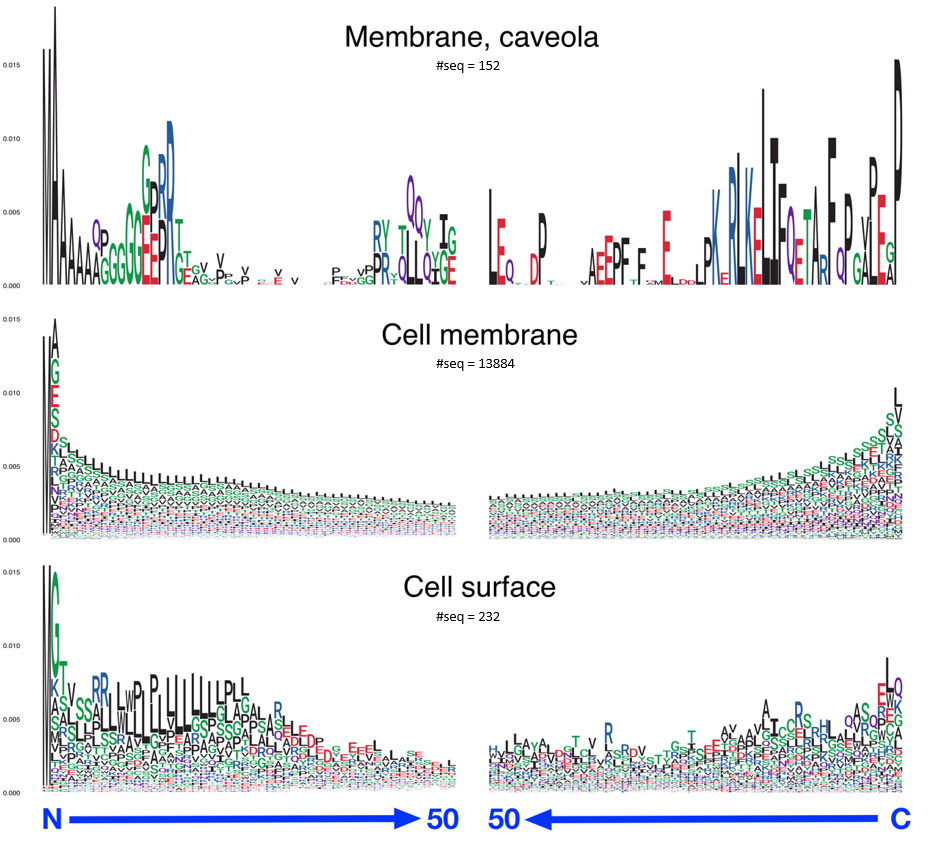


**Figure S11-2**. The attention weights at the N-terminus (left column) and the C-terminus (right column) for protein localizations under the membrane category.


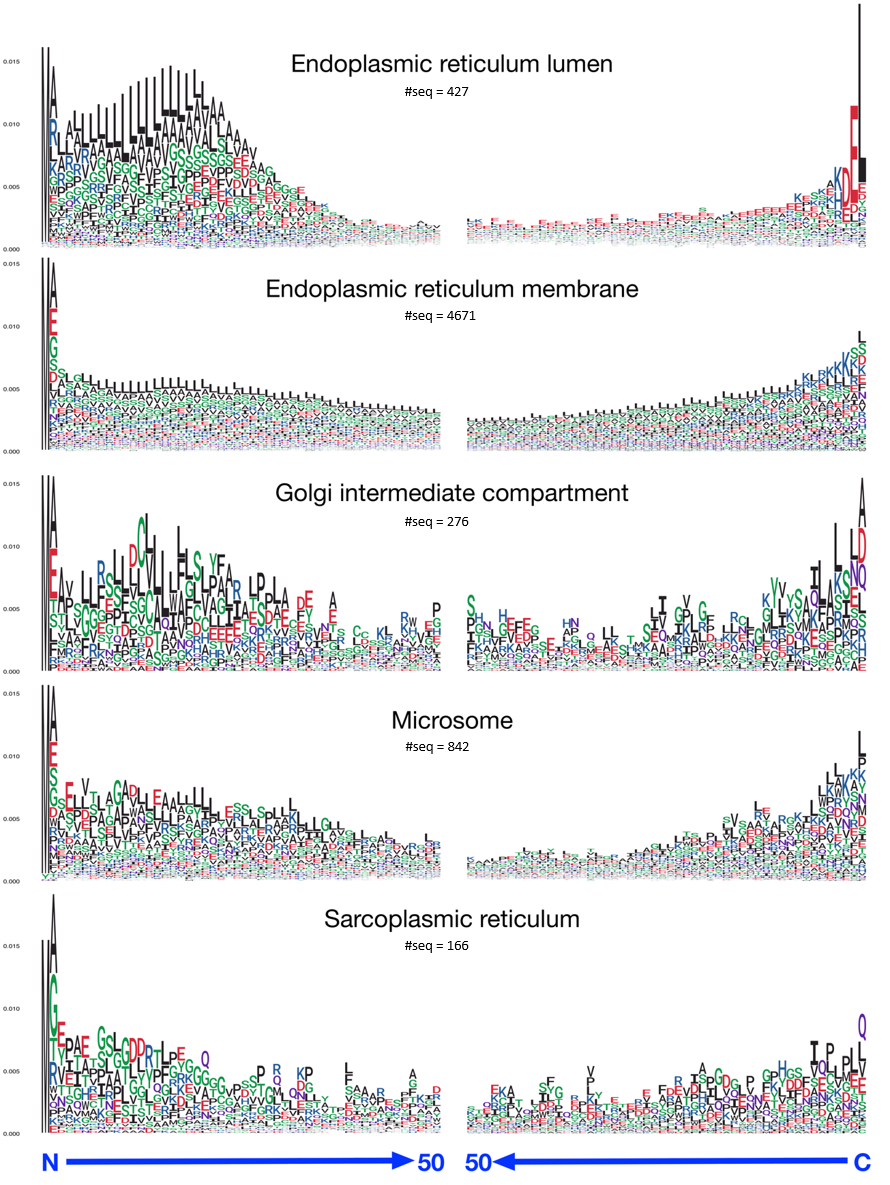


**Figure S12**. The attention weights at the N-terminus (left column) and the C-terminus (right column) for protein localizations under the endoplasmic reticulum organelle.


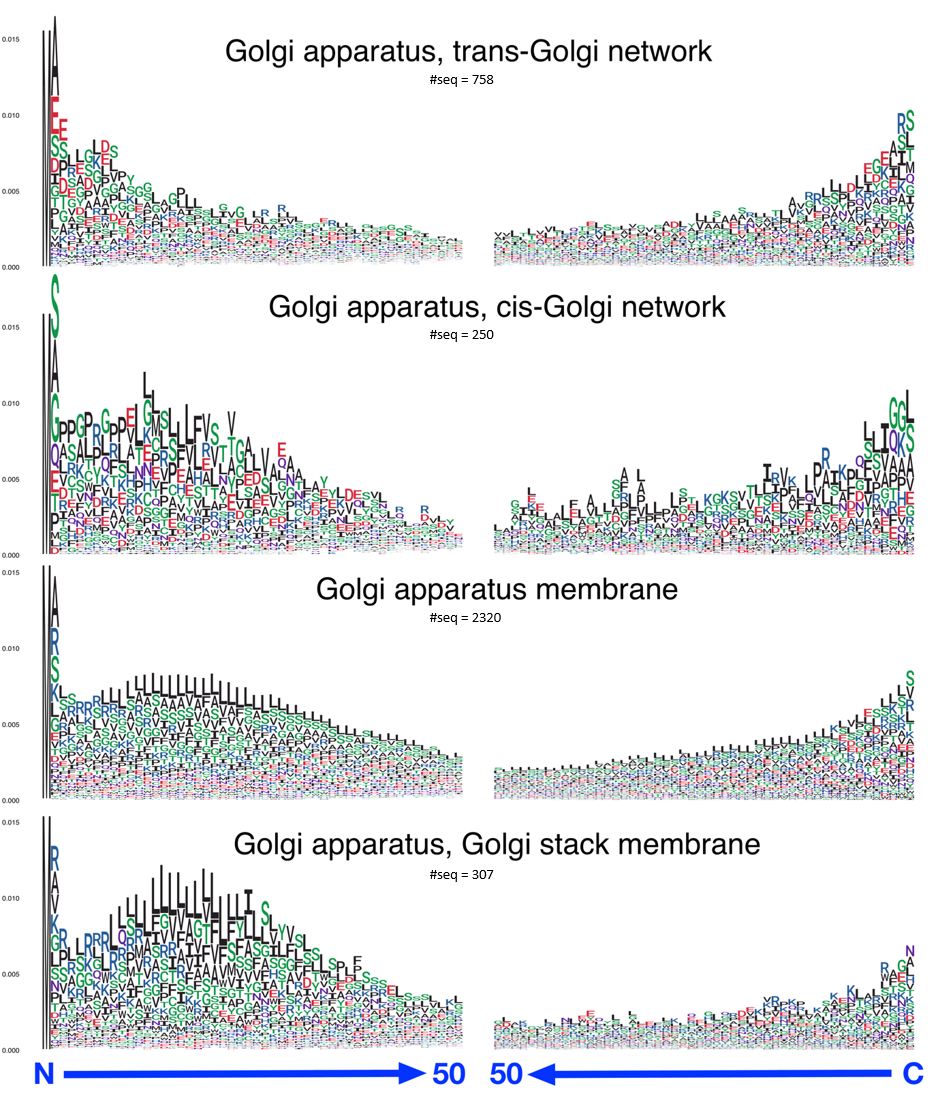


**Figure S13**. The attention weights at the N-terminus (left column) and the C-terminus (right column) for protein localizations under the Golgi apparatus organelle.


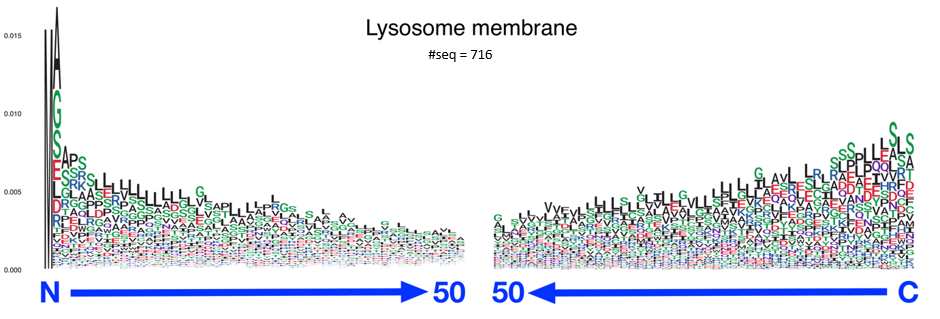


**Figure S14**. The attention weights at the N-terminus (left column) and the C-terminus (right column) for protein localizations under the lysosome/vacuole organelle.


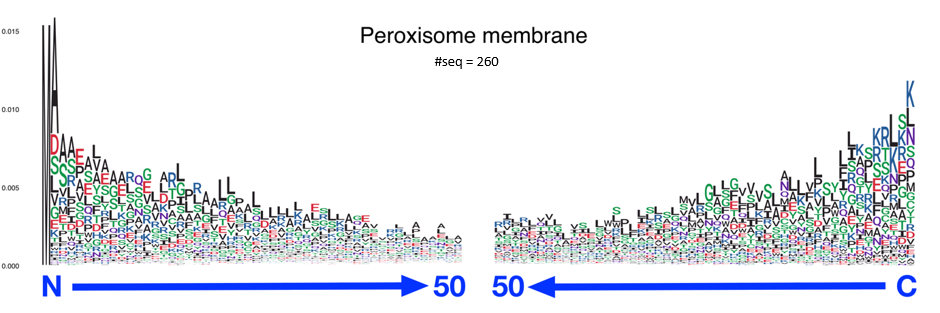


**Figure S15**. The attention weights at the N-terminus (left column) and the C-terminus (right column) for protein localizations under the peroxisome organelle.

| Localization | Rank | Motif | |
| --- | --- | --- | --- |
| Cytoplasm | 1  (480) | logo | 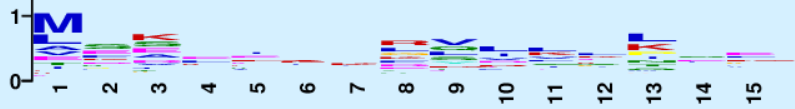 |
|  |  | RE | MX-XXX-X-X-X-X-X-X-XXX-XX |
|  | 2  (-2023) | logo | 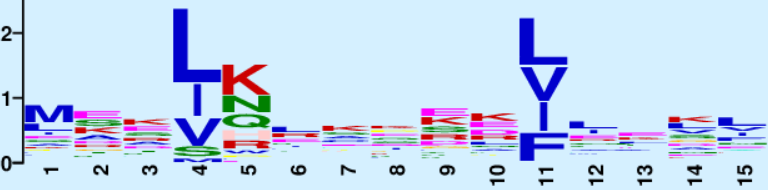 |
|  |  | RE | M-X-X-X-I/L-K-XX-X-X-X-F/I/L/V-X-X-XL |
|  | 3  (-2247) | logo | 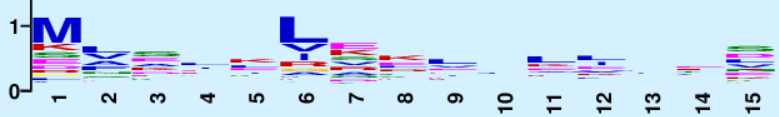 |
|  |  | RE | M-X-XXXX-L-X-X-XX-X-X-L-X-X-X-X-X- |

**Figure S16**. The top 3 GLAM2 results for proteins in the cytoplasm. For each result, its rank, score, sequence logo, and the regular expression (RE) of the motif are given.

**Figure S17**. The top 3 GLAM2 results for proteins in the cell membrane. For each result, its rank, score, sequence logo, and the regular expression (RE) of the motif are given.

| Localization | Rank | Motif | |
| --- | --- | --- | --- |
| Cell membrane | 1  (8919) | logo | 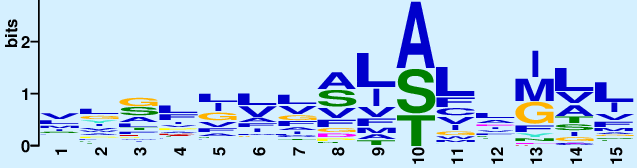 |
|  |  | RE | XV-XX-XLLVVA/I-LL-XLLF- |
|  | 2  (8664) | logo | 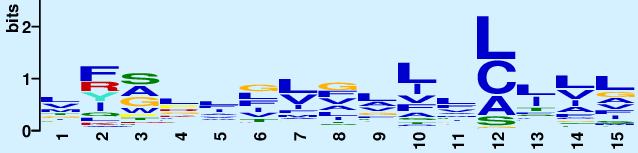 |
|  |  | RE | XF-S-L-X-XL/VX-L-X-L-LC/L-I/L-LX-L- |
|  | 3  (3442) | logo | 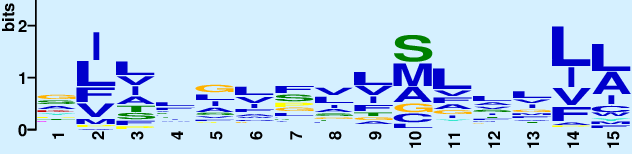 |
|  |  | RE | XX-I/L-X-LX-XXLX-V-LM/S-LL-I/LA/L- |

**Figure S18**. The top 3 GLAM2 results for proteins in the endoplasmic reticulum. For each result, its rank, score, sequence logo, and the regular expression (RE) of the motif are given.

| Localization | Rank | Motif | |
| --- | --- | --- | --- |
| Endoplasmic reticulum | 1  (6019) | logo | 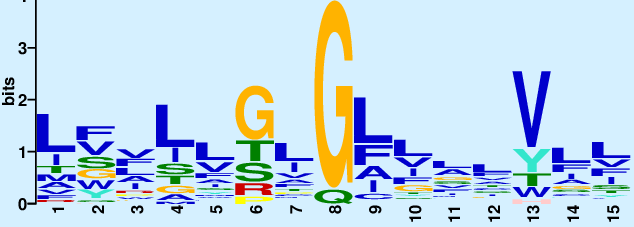 |
|  |  | RE | LX-XL-LG-LG-F/LLXLV-LL |
|  | 2  (3129) | logo | 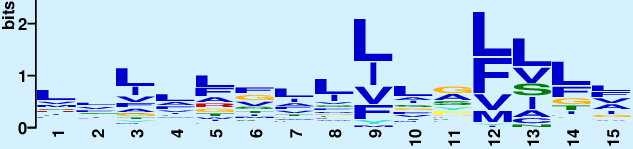 |
|  |  | RE | LX-L-LX-L-X-LLIL-LX-X-F/L-L-L-X- |
|  | 3  (2986) | logo | 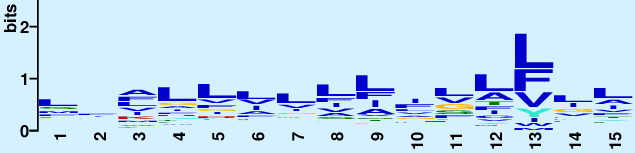 |
|  |  | RE | L-XX-LL-LLLL-X-X-X-LL-F/LLL- |

**Figure S19**. The top 3 GLAM2 results for proteins in the Golgi apparatus. For each result, its rank, score, sequence logo, and the regular expression (RE) of the motif are given.

| Localization | Rank | Motif | |
| --- | --- | --- | --- |
| Golgi apparatus | 1  (2260) | logo | 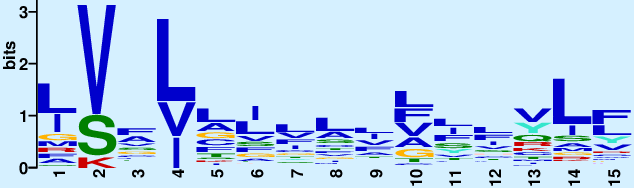 |
|  |  | RE | I/LS/V-XI/L/V-LI/LLLXLXXV-LF |
|  | 2  (2208) | logo | 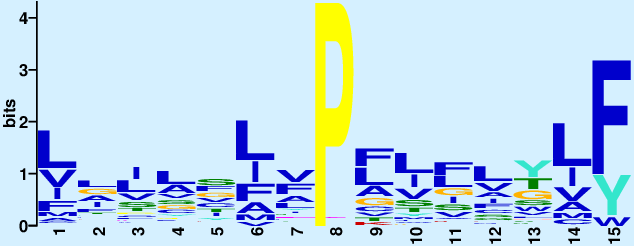 |
|  |  | RE | L.[IL]L.?[IL][FV]?P?[FL]LF?LY?L[YF]?  LXI/LLX-I/LF/V-P-F/LLF-LY-LY/F- |
|  | 3  (2166) | logo | 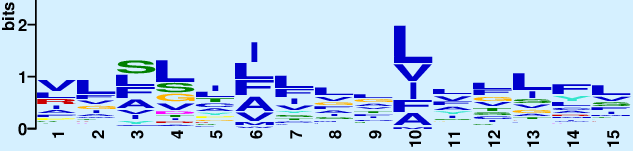 |
|  |  | RE | VLSLX-L-LLXLX-XLFL |

**Figure S20**. The top 3 GLAM2 results for proteins in the lysosome. For each result, its rank, score, sequence logo, and the regular expression (RE) of the motif are given.

| Localization | Rank | Motif | |
| --- | --- | --- | --- |
| Lysosome | 1  (1574) | logo | 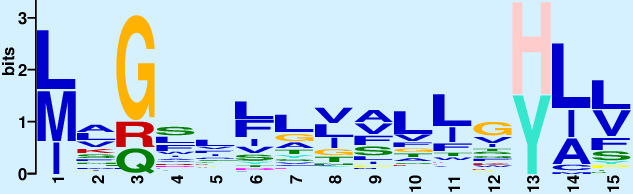 |
|  |  | RE | I/M/L-XG-SLF/LLL/V-ALI/LG-Y/H-I/A/L-L/V |
|  | 2  (913) | logo | 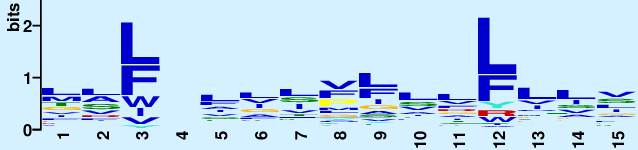 |
|  |  | RE | X-XF/L-X-L-XX-X-LX-LLF/LLLX |
|  | 3  (727) | logo | 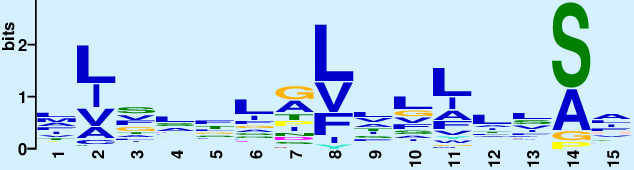 |
|  |  | RE | XX-I/L-X-L-XL-A/G-L/V-XLL-LXA/S-X |

**Figure S21**. The top 3 GLAM2 results for proteins in the peroxisome. For each result, its rank, score, sequence logo, and the regular expression (RE) of the motif are given.

| Localization | Rank | Motif | |
| --- | --- | --- | --- |
| Peroxisome | 1  (636) | logo | 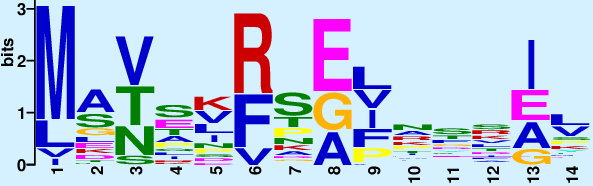 |
|  |  | RE | MAN/T/V-SK-R/F-SA/E/G-L-XXXI/A/E-L/V |
|  | 2  (613) | logo | 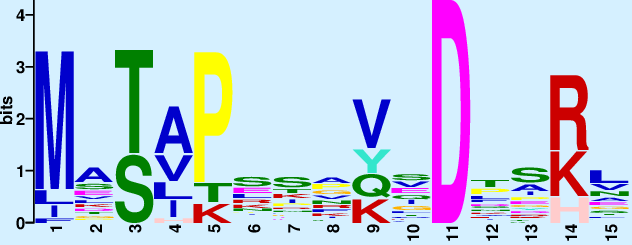 |
|  |  | RE | MAS/T-A/V-P-SSXQ/Y/K/V-X-D-XSK/R-L |
|  | 3  (543) | logo | 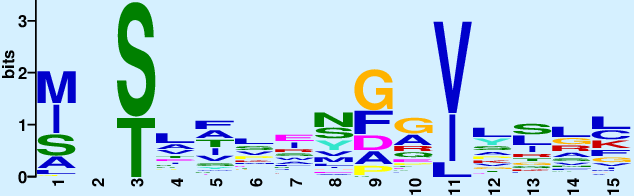 |
|  |  | RE | I/M/SX-S/T-LX-XXN-G/F-GI/V-LSLL |

**Supplementary Note 1: Training process pseudo code**

Algorithm 1 Train MULocDeep model

Input: UniLoc_train dataset, predefined hyperparameters, MULocDeep model with untrained parameters $\theta$.

1: $\left[ \left( train_{1}, evaluation_{1} \right), \left( train_{2}, evaluation_{2} \right),\ldots, \left( train_{8}, evaluation_{8} \right) \right]\leftarrow split(UniLoc\_train)$

2: for f = 1 to 8 do

3: initial $model_{f}$ with random parameters $\theta$

4： $\left[ \left( lv1\_trainX_{f}, lv1\_trainY_{f} \right), \left( lv2\_trainX_{f}, lv2\_trainY_{f} \right) \right]\leftarrow from train_{f}$

5: $\left( lv1\_valX_{f}, lv1\_valY_{f} \right)\leftarrow from evaluation_{f}$

6: for epoch = 1 to 80 do

$\mathcal{L}_{lv1}\mathcal{\leftarrow L(}model_{f}\left( encode\left( lv2\_trainX_{f} \right);\theta\right), lv2\_trainY_{f})$

10: $\mathcal{L}_{lv2}\mathcal{\leftarrow L(}model_{f}\left( encode\left( lv2\_trainX_{f} \right);\theta\right), lv2\_trainY_{f})$

11: $Update \theta using \frac{\delta(\mathcal{L}_{lv1}+\mathcal{L}_{lv2})}{\delta\theta}$

7: $\mathcal{L}_{lv1}\mathcal{\leftarrow L(}model_{f}\left( encode\left( lv1\_trainX_{f} \right);\theta\right), lv1\_trainY_{f})$

8: $ACC_{lv1}\leftarrow ACC(model_{f}\left( encode\left( lv1\_valX_{f} \right); \theta\right), lv1\_valY_{f})$

9: $Update \theta using \frac{\delta\mathcal{L}_{lv1}}{\delta\theta}$

12: If $ACC_{lv1}$ > $ACC_{lv1} from last epoch$

13: Save $model_{f}$

14: end for

15: end for

16: MULocDeep $\leftarrow$ ensemble ($model_{1}, model_{2}, \ldots, model_{8}$)

**Supplementary Note 2: Variant model and the evaluation results**

The architecture of a variant MULocDeep model is shown in Figure S3. It is the same as the MULocDeep model before the fully connected layer (1 by 80) in Figure 1. The difference is that instead of reshaping the vector into a matrix in Figure 1, we used the vector as output directly for the variant model. The length of the vector is adjusted according to the method we compared with, either to predict subcellular localizations (equivalent to one row in the matrix in Figure 1) or to predict suborganellar localizations under one specific organelle (equivalent to one column in the matrix in Figure 1). Thus, when training a variant model, we only used the corresponding training data provided by the specific methods that we wanted to compare with. The variant model also used the same final optimized hyperparameters as in the MULocDeep model. We also needed to pick the combination of either a softmax activation function with a categorical cross entropy loss function, or a sigmoid activation function with a binary cross entropy loss function, based on the situation if the other method was a single-label or multi-label prediction method, respectively.

**Supplementary Note 3: Mitochondrial proteome identification**

**Mitochondria isolation from *Arabidopsis thaliana* cell cultures**

Arabidopsis cell cultures were cultivated and mitochondria were isolated as described by Werhahn *et al*., (1) with slight modifications. In brief, cells were filtered through four layers of gauze and disrupted in a Waring Blender in disruption buffer (450 mM sucrose; 15 mM MOPS; pH 7.4 [KOH]; 0.6% [w/v] PVP 40; 1.5 mM EGTA; 0.2% [w/v] BSA; 10 mM sodium ascorbate; 10 mM cysteine; 0.2 mM PMSF) for 15 seconds at full speed and twice for 15 s at the lowest possible speed. The homogenate was first centrifuged at 2700 × g for 5 minutes, followed by another centrifugation of the supernatant at the same settings. The resulting supernatant was then centrifuged at 8300 × g for 5 minutes. Crude mitochondria were pelleted by centrifuging the supernatant at 17,000 × g for 10 minutes. The pellet was resuspended in 3-5 ml wash buffer (300 mM sucrose; 10 mM MOPS, pH 7.2 [KOH]; 1 mM EGTA; 0.2 mM PMSF), subjected to two strokes in a Dounce homogenizer and layered on top of discontinuous Percoll (GE Healthcare, Uppsala, Sweden) gradients (18%/23%/40% [v/v] Percoll in 300 mM sucrose; 10 mM MOPS, pH 7.2 [KOH]), which were centrifuged at 70000 x g for 90 minutes. Mitochondria formed a layer at the approximate position of the original border between the 40% and 23% Percoll phase, which was removed, diluted in a resuspension buffer (400 mM mannitol; pH 7.2 [KOH]; 1 mM EGTA; 10 mM Tricine; 0.2 mM PMSF), and centrifuged at 14500 × g for 10 minutes. The remaining Percoll in the mitochondrial fraction was removed by repeatedly resuspending the pellets in a resuspension buffer, followed by centrifugation and the removal of the supernatant.

**Mitochondria isolation from Solanum tubers**

Mitochondria were isolated according to Bultema *et al*. (2) and Neuburger *et al*. (3) with slight modifications. 1.2 kg potato tubers were peeled and disrupted in a Waring Blender in disruption buffer (400 mM mannitol; 25 mM MOPS; pH 7.8 [KOH]; 1 mM EGTA; 15 mM β-mercaptoethanol; 0.05 mM PMSF; 0.1% [w/v] BSA). The pulp was filtered through two layers of muslin and centrifuged at 3500 × g for 5 min. The supernatant was centrifuged at 17,000 × g for 30 minutes. The starch-containing phase of the pellet was resuspended and discarded. The remaining pellet was resuspended resuspension buffer (400 mM mannitol; pH 7.2 [KOH]; 1 mM EGTA; 10 mM potassium phosphate; 0.2 mM PMSF) and homogenized with two strokes in a Dounce homogenizer. The homogenate was layered on top of a discontinuous Percoll gradient (14/26/45%) in resuspension buffer. The purification of the mitochondria was achieved using centrifugation at 70,000 × g for 45 minutes. The mitochondrial layer between the 45% and 26% Percoll layer was removed with a glass pipette. The remaining Percoll was removed by 2-3 centrifugation steps at 14,500 × g for 10 minutes. Between each centrifugation step the pellet was diluted using a resuspension buffer, which was also used to resuspend the final mitochondrial pellet.

**Mitochondria isolation from *Solanum tubers* with subsequent iTRAQ labelling**

The isolation procedure was executed according to Havelund *et al*. (4). One kilogram of potato tubers were peeled and homogenized in a juice extractor and diluted in a ratio of 2:1 with extraction medium (900 mM mannitol; 30 mM MOPS; pH 7.3 [KOH]; 3 mM EDTA; 25 mM cysteine; 0.3% [w/v] BSA). The pH was immediately adjusted with KOH to 7.2. The homogenate was incubated for 5 minutes and filtered through two layers of cotton and centrifuged at 3000 × g for 5 minutes. The pellets were discarded and the supernatants were centrifuged again at 18000 x g for 10 minutes. The resulting pellets were resuspended in gradient buffer (300 mM mannitol; 10 mM MOPS; pH 7.2 [KOH]; 0.1% [w/v] BSA) and layered on top of a discontinuous Percoll gradient consisting of a 20%, 28%, and 50% Percoll layer in a gradient buffer. The gradient was centrifuged at 40,000 x g for 30 minutes. The mitochondrial band was collected, filled up with wash buffer (300 mM mannitol; 10 mM MOPS; pH 7.2 [KOH]; 1 mM EDTA), and the remaining Percoll was removed using 2 washing steps at 18,000 × g for 10 minutes. In between the supernatant was discarded, and the tube was filled up with fresh wash buffer. The pellets were resuspended in gradient buffer layered on top of the 28% Percoll gradient phase and centrifuged at 40,000 × g for 30 minutes. The purified mitochondrial band was transferred to new tubes, filled up with wash buffer, and washed twice at 18,000 × g for 10 minutes. The final pellet was resuspended in 500 µL wash buffer. The mitochondria were pelleted again at 14000 × g for 5 minutes and disrupted in lysis buffer (4% [w/v] SDS; 20 mM triethylammonium bicarbonate; pH 8.5; 0.1 M DTT). The proteins were on-filter digested according to Leon *et al*., (5) and iTRAQ-labelled according to the manufacturer’s instructions (AB Sciex).

**Mitochondria isolation from *Vicia faba* roots**

The isolation protocol was adapted from Bultema *et al*. (2) with some modifications. *Vicia faba* plants were grown in a short-day (8 hours) light regime. Once a week, the plants were fertilized with 0.5x Hoagland’s fertilizer (Hoagland *et al*. (6)). 50 g roots of 5-week old plants were ground using a mortar and pestle in 100 mL disruption buffer (400 mM mannitol; 25 mM MOPS, pH 7.8 [KOH]; 1 mM EGTA; 5 mM cysteine; 0.1% [w/v] BSA; 0.05 mM PMSF). The homogenate was filtered through 4 layers of Miracloth and centrifuged at 400 × g for 5 minutes. The pellet was discarded and the supernatant was centrifuged at 4000 x g for 5 minutes. The supernatant was centrifuged at 17000 × g for 20 minutes and the pellet with the crude mitochondria was resuspended in 6 mL of resuspension buffer (400 mM mannitol; pH 7.2 [KOH]; 1 mM EGTA; 10 mM potassiumphosphate; 0.05 mM PMSF). The suspension was homogenized with 5 strokes in a Dounce homogenizer and loaded onto a discontinuous Percoll gradient consisting of 14%, 22%, and 45% Percoll layer (in resuspension buffer). The crude mitochondria were purified at 35000 × g for 30 minutes. The mitochondrial band between 22% and 45% was removed with a glass pipette and diluted with resuspension buffer. The remaining Percoll was removed with 2 washing steps at 18000 × g for 10 minutes and one final centrifugation at 10000 × g for 10 minutes. The purified mitochondria were taken up in a resuspension buffer.

**Protein extraction and mass spectrometry**

The mitochondrial proteins of the three species, except for iTRAQ labelled samples, were identified and quantified using gel-based shotgun mass spectrometry according to Thal *et al*. (7). The protein concentration of the isolated mitochondria of the three species was determined using the Bradford method.(8) A volume equivalent to that of a 50 µg protein was mixed with loading buffer (62.5 mM Tris; pH 6.8 [HCl]; 2% [w/v] SDS; 10% [v/v] glycerol; 5% [v/v] β-mercaptoethanol) and incubated at 60° C for 5 minutes. Solubilized proteins were loaded on a SDS-gel that consisted of a 4% [w/v] acrylamide top phase (pH 6.8) and a 14% [w/v] acrylamide (pH 8.8) bottom phase, respectively. The gel run was stopped when the samples formed a focused line at the transition between the top and bottom phases. The proteins were fixated to the gel matrix by incubation in 15% [v/v] ethanol and 10% [v/v] acetic acid, followed by staining with Coomassie Brilliant Blue G250 (9). The stained gel bands were excised and digested in-gel according to Fromm *et al*. (10). The resulting peptides were extracted from the gel matrix and separated using liquid chromatography in an Ultimate 3000 (Thermo Fisher Scientific, Dreieich, Germany) UPLC to be subsequently analyzed using a Q-Exactive (Thermo Fisher Scientific, Dreieich, Germany) as described in Fromm *et al*. (10). iTRAQ labelled peptides were lyophilized and quantified via liquid-tandem mass spectrometry according to Havelund. (4)

**Mass spectrometry raw data processing**

Raw files were analyzed with the Proteome Discoverer software (Thermo Fisher Scientific, Dreieich, Germany) using the Mascot (Matrix Science, London, UK) search engine and a peptide database depending on the analyzed species: Arabidopsis, Solanum, and Vicia peptides were queried against the TAIR 10 database, an in-house Solanum protein database, and the Medicago protein database 4.0, respectively. The settings for the selection of spectra were as follows: Precursor selection, Use MS1 Precursor; Use new Precursor Reevaluation, True; Lower and upper RT limit, 0; First and last scan, 0; Lowest and highest charge state, 0; Min. precursor mass, 350 Da; Max. precursor mass, 5000 Da; Total intensity threshold, 0; Minimum peak count, 1; Mass analyzer, Is FTMS; MS order, Is MS2; Activation type, Is HCD; Min. collision energy, 0; Max. collision energy, 1000; Scan type, Is full; S/N threshold, 1.5; Unrecognized charge replacements, Automatic; Unrecognized mass analyzer replacements, FTMS; Unrecognized MS order replacements, MS2; Unrecognized activation type replacements, HCD; Unrecognized polarity replacements, +. The settings for the Mascot algorithm were: Enzyme name, Trypsin; Maximum missed cleavage sites, 2; Instrument, Default; Taxonomy, All entries; Peptide cut off score, 10; Peptide without protein cut off score, 5; Use MudPIT scoring, automatic; Protein relevance threshold, 20; Protein relevance factor, 1; Precursor mass tolerance, 10 ppm; Fragment mass tolerance, 0.05 Da; Use average precursor mass, false; Dynamic modifications, acetyl (N-term), oxidation (M), deaminated (NQ); Static modifications, Carbamidomethyl (C). The false discovery rate (FDR) was set to 0.01. The settings for the iTRAQ labelled samples were identical, except for the detected modifications: From quantification method, iTRAQ 4plex (protein); Dynamic modifications, oxidation (M), deaminated (NQ); Static modifications, carbamidomethyl (C).

**References**

1. Werhahn, W., Niemeyer, A., Jänsch, L., Kruft, V., Schmitz, U.K. and Braun, H.-P. (2001) Purification and characterization of the preprotein translocase of the outer mitochondrial membrane from Arabidopsis. Identification of multiple forms of TOM20. *Plant Physiology*, **125**, 943-954.

2. Bultema, J.B., Braun, H.P., Boekema, E.J. and Kouril, R. (2009) Megacomplex organization of the oxidative phosphorylation system by structural analysis of respiratory supercomplexes from potato. *Biochim Biophys Acta*, **1787**, 60-67.

3. Neuburger, M., Journet, E.-P., Bligny, R., Carde, J.-P. and Douce, R. (1982) Purification of plant mitochondria by isopycnic centrifugation in density gradients of Percoll. *Archives of Biochemistry Biophysics*, **217**, 312-323.

4. Havelund, J.F., Salvato, F., Chen, M., Rao, R.S.P., Rogowska-Wrzesinska, A., Jensen, O.N., Gang, D.R., Thelen, J.J. and Møller, I.M. (2014) Isolation of mitochondria from potato tubers. *Bio-protocol*, **4**, e1226.

5. León, I.R., Schwämmle, V., Jensen, O.N. and Sprenger, R.R. (2013) Quantitative assessment of in-solution digestion efficiency identifies optimal protocols for unbiased protein analysis. *Molecular Cellular Proteomics*, **12**, 2992-3005.

6. Hoagland, D.R. and Arnon, D.I. (1950) The water-culture method for growing plants without soil. *Circular. California agricultural experiment station*, **347**.

7. Thal, B., Braun, H.P. and Eubel, H. (2018) Proteomic analysis dissects the impact of nodulation and biological nitrogen fixation on Vicia faba root nodule physiology. *Plant molecular biology*, **97**, 233-251.

8. Bradford, M.M. (1976) A rapid and sensitive method for the quantitation of microgram quantities of protein utilizing the principle of protein-dye binding. *Anal Biochem*, **72**, 248-254.

9. Neuhoff, V., Stamm, R. and Eibl, H. (1985) Clear background and highly sensitive protein staining with Coomassie Blue dyes in polyacrylamide gels: a systematic analysis. *Electrophoresis*, **6**, 427-448.

10. Fromm, S., Senkler, J., Eubel, H., Peterhänsel, C. and Braun, H.-P. (2016) Life without complex I: proteome analyses of an Arabidopsis mutant lacking the mitochondrial NADH dehydrogenase complex. *Journal of experimental botany*, **67**, 3079-3093.
